# Toll-like Receptor 4 Deficiency Reduces Oxidative Stress and Macrophage Mediated Inflammation in Hypertensive Kidney

**DOI:** 10.1038/s41598-017-06484-6

**Published:** 2017-07-25

**Authors:** Sathnur Pushpakumar, Lu Ren, Sourav Kundu, Alejandra Gamon, Suresh C. Tyagi, Utpal Sen

**Affiliations:** 1 Department of Physiology, University of Louisville School of Medicine, Louisville, KY-40202 USA; 2 Institute of Advanced Study in Science and Technology, Guwahati, Assam 781035 India; 3 University of California, San Diego, CA 92093 USA

## Abstract

Oxidative stress and inflammation are integral to hypertension-induced renal injury. A unifying feature for the two components is Toll-like receptors (TLR), which are key regulators of the innate immune system. Recent studies implicate TLR4 activation and oxidative stress in cardiovascular diseases and also as a link between inflammation and hypertension. However, its role in hypertension induced renal injury remains unexplored. In the present study, we investigated whether TLR-4 deficiency reduces Ang-II-induced renal injury and fibrosis by attenuating reactive oxygen species (ROS) production and inflammation. C3H/HeOuJ mice with normal TLR-4 and C3H/HeJ^*Lps-d*^ with dysfunctional TLR4 (TLR4 deficiency) were treated without or with Ang-II. In response to Ang-II, TLR4 deficient mice had reduced renal resistive index and increased renal cortical blood flow compared to mice with normal TLR4. Further, TLR4 deficiency reduced oxidative stress and increased antioxidant capacity (MnSOD, CuSOD and Catalase activity). TLR4 deficiency was also associated with reduced inflammation (MCP-1, MIP-2, TNF-α, IL-6 and CD68), decreased accumulation of bone marrow-derived fibroblasts and TGF-β expression. Our data suggests that in C3H/HeJ^*Lps-d*^ mice, deficiency of functional TLR4 reduces oxidative stress and macrophage activation to decrease TGF-β-induced extracellular matrix protein deposition in the kidney in Ang-II induced hypertension.

## Introduction

Hypertension is the second leading cause of chronic kidney disease (CKD) in the world. At the subcellular level, oxidative stress and inflammation are two critical components in the pathogenesis of hypertension-induced organ damage^1^. Reactive oxygen species (ROS) are by-products of oxidative phosphorylation in the mitochondria and other oxidoreductase reactions and commonly include superoxide (O_2_^−•^), hydrogen peroxide (H_2_O_2_) and hydroxyl anions (OH^−•^)^2^. Excess ROS can result in impairment of redox signaling pathways leading to cellular damage and dysfunction. The deleterious effects of ROS are normally countered by an effective anti-oxidant system. An imbalance in the production of ROS and its breakdown known as oxidative stress can be a cause and consequence of hypertension. In the kidney, the interaction between superoxide and nitric oxide causes damage to the vascular endothelium promoting vasoconstriction^3^. Further, high levels of Angiotensin-II (Ang-II) increase ROS via upregulation of NADPH oxidase system which result in infiltration of inflammatory cells causing glomerular and tubular damage^1^.

The inflammatory response can precede or follow the onset of hypertension and is directly related to oxidative stress. Cross-sectional studies have shown that inflammatory markers are elevated in the serum of pre-hypertensive subjects and patients with established hypertension^4, 5^. Recent studies have shown that macrophages play an important role in hypertension induced kidney injury^6, 7^. In spontaneously hypertensive rats, accumulation of macrophages and lymphocytes has been documented in the kidneys even before the onset of hypertension^8^. The inflammatory cells are known to localize in the perivascular and glomerular regions of the kidney^9^.

Toll-like receptors are part of the innate immune system which responds to factors derived from pathogens or cellular damage to elicit an effective defense^10^. Endogenous molecules termed damage associated molecular patterns are believed to activate Toll-like Receptors (TLRs) to initiate an inflammatory response in hypertension. Studies have demonstrated Toll-like Receptor 4 (TLR4) activation in ischemia reperfusion injury and haemorrhagic shock which are associated with oxidative stress^11, 12^. TLR4 has also been implicated in the development and progression of cardiovascular diseases by inducing oxidative stress and endothelial dysfunction^13, 14^. TLR4 activation has also been reported to cause oxidative stress and vascular injury following Angiotensin-II (Ang-II) infusion^15^. In renal pathologies, TLR4 mediated inflammation has been studied in unilateral ureteral obstruction^16^, interstitial nephritis^17^, diabetic nephropathy^18^, and ischemia reperfusion injury^19^. More recently, TLR4 activation was found to mediate proinflammatory response in cyclosporine induced nephrotoxicity and inhibition of TLR4 by treatment with TAK242 was shown to abrogate renal injury and fibrosis in this model^20^. The purpose of our study was to determine 1) the role of TLR4 in oxidative stress induction in the hypertensive kidney, 2) whether TLR4 induced oxidative stress leads to macrophage recruitment and inflammation, 3) whether TLR4 deficiency abrogates the effects above and inhibits fibroblast accumulation to reduce renal fibrosis.

## Results

### TLR4 deficiency blunts the effect of Ang-II on hypertension, renal blood flow, vascular resistance and reduces renal injury

**Figure 1:**
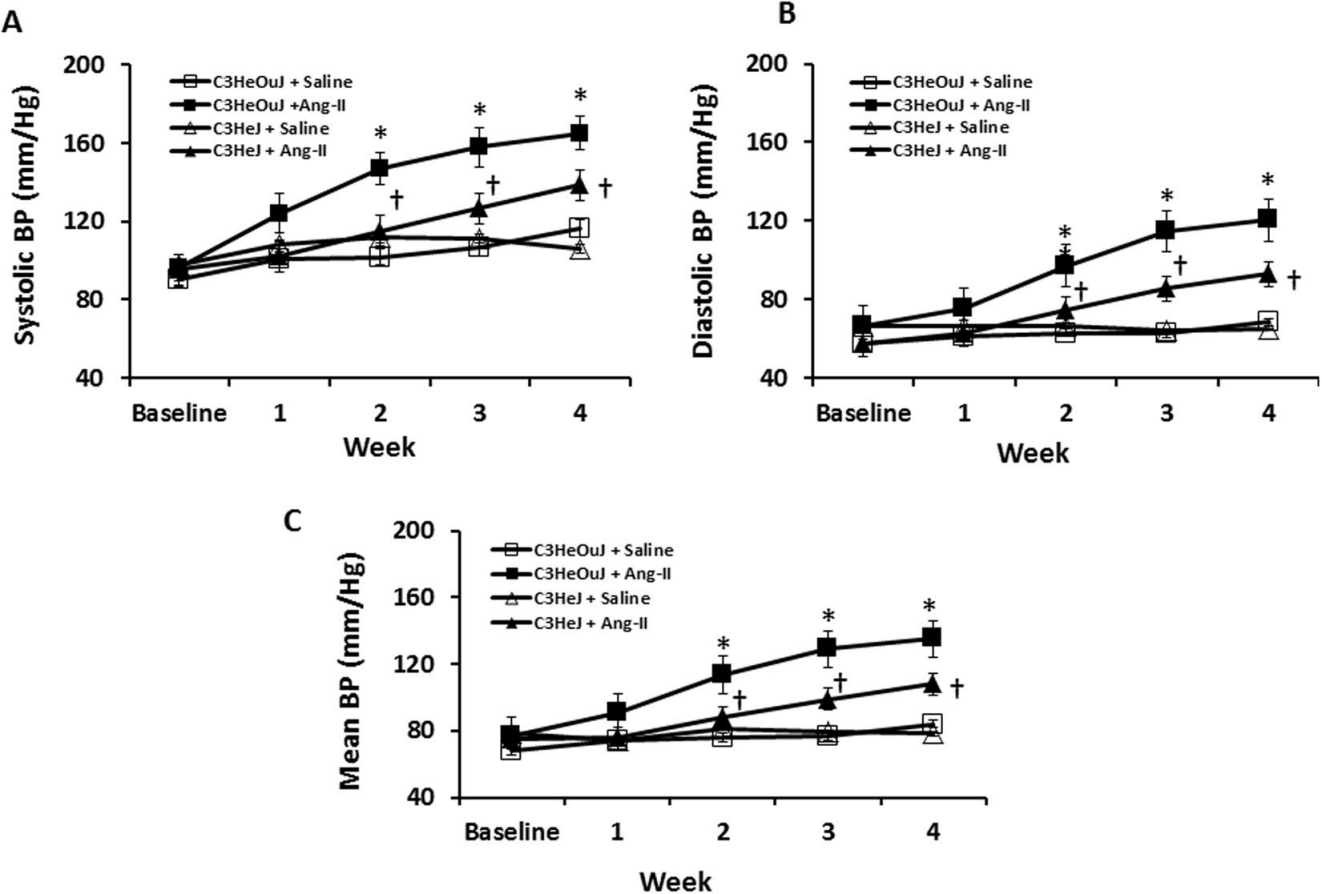
TLR4 deficiency mice exhibit blunted response to Ang-II induced hypertension. Time course of systolic, diastolic and mean blood pressure changes following Ang-II treatment in C3H/HeOuJ (C3HeOuJ, normal TLR4) and C3H/HeJ (C3HeJ, TLR4 deficiency) mice. n = 7/group tested by ANOVA followed by Student’s t-test. *p < 0.05 vs. Saline groups; ^†^p < 0.05 vs. C3HeOuJ + Ang-II.

**Figure 2:**
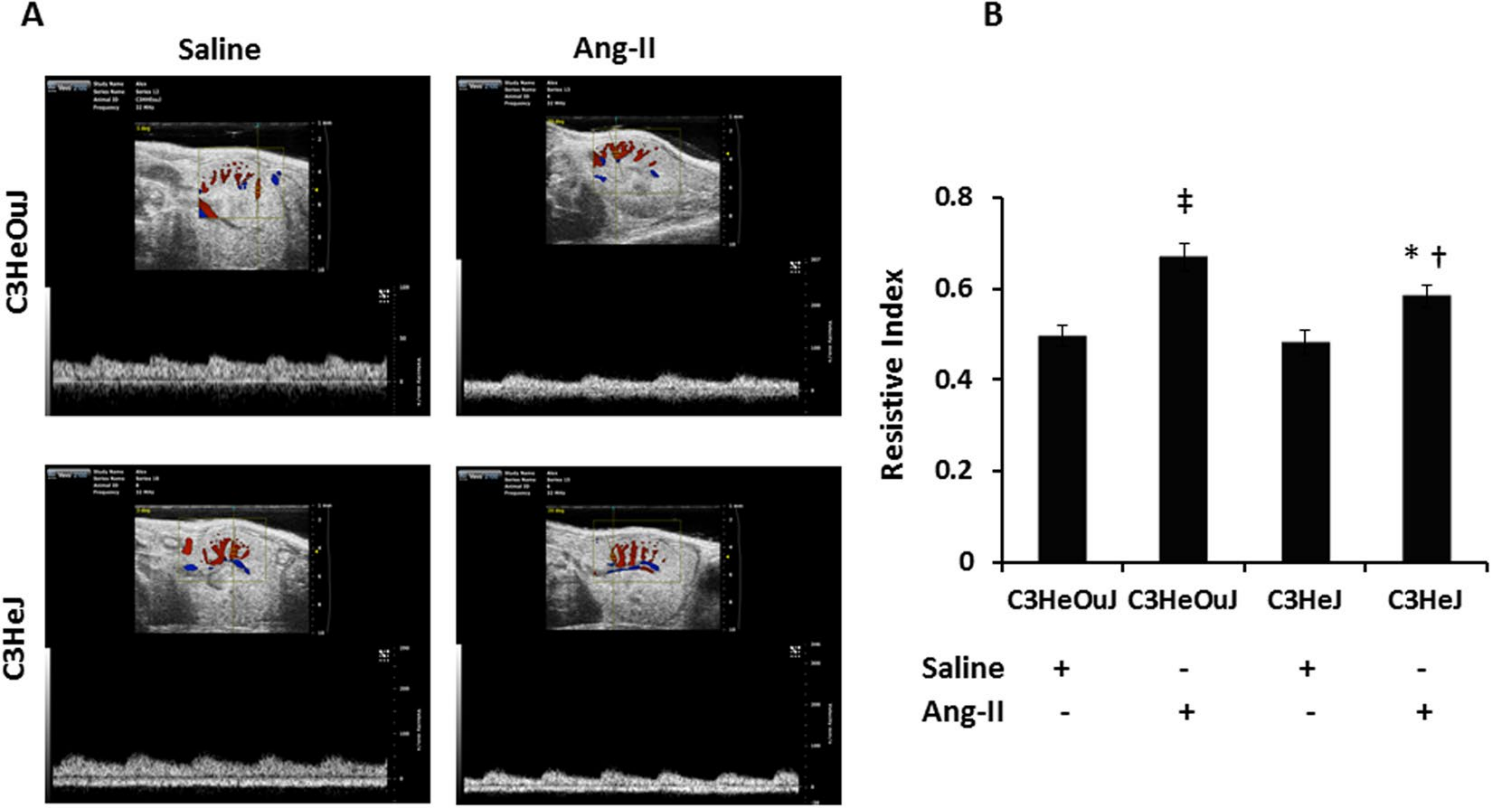
The resistive index of intrarenal cortical artery is decreased in TLR4 deficiency mice to Ang-II. (**A**) Representative images of left kidney in short axis. The transducer is fixed to obtain a window showing cortical vessels. The pulse wave cursor is then centered on a cortical artery with its axis in parallel and vascular signals are obtained. Resistive index is calculated by the formula (PSV-EDV)/PSV. PSV, peak systolic velocity; EDV, end diastolic velocity. (**B**) Bar graph shows mean resistive index ± SEM. n = 6/group tested by ANOVA and Student’s t-test. *p < 0.05 vs. C3HeJ + saline, ^†^p < 0.05 vs. C3HeOuJ + Ang-II, ^‡^p < 0.05 vs. C3HeOuJ + saline. C3HeOuJ: normal TLR4, C3HeJ: TLR4 deficiency.

**Figure 3:**
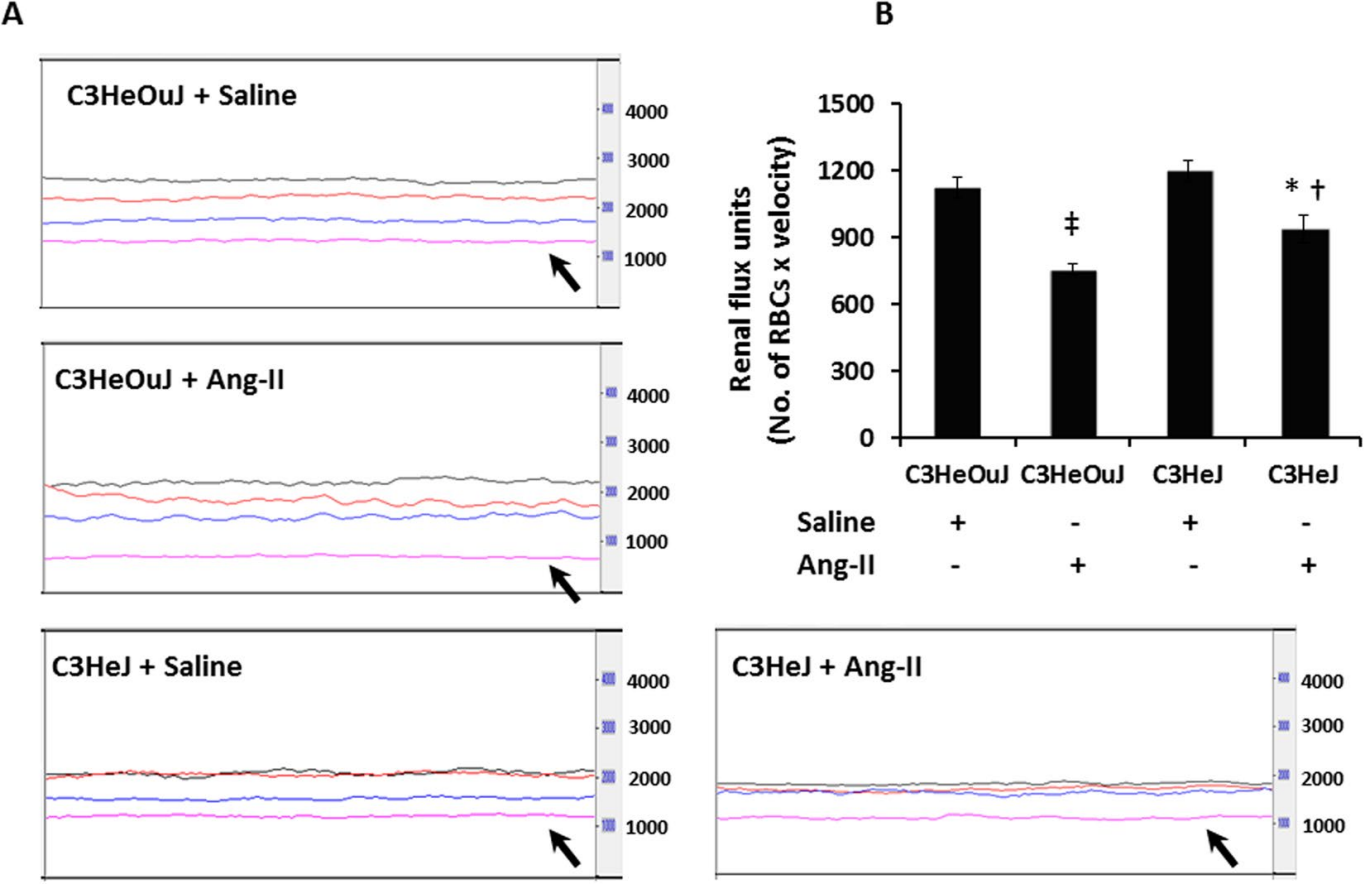
TLR4 deficiency mice maintain better renal cortical blood flux than mice with normal TLR4 in response to Ang-II treatment. (**A**) Representative line tracing of aorta (black), renal artery (red), renal vein (blue) and renal cortex (pink). (**B**) Data shows mean flux ± SEM. n = 6/group tested by ANOVA and Student’s t-test. RBC, red blood cell. *p < 0.05 vs. C3HeJ + saline, ^†^p < 0.05 vs. C3HeOuJ + Ang-II, ^‡^p < 0.05 vs. C3HeOuJ + saline. C3HeOuJ: normal TLR4, C3HeJ: TLR4 deficiency.

There was no difference in blood pressure (BP) in mice receiving saline treatment. In C3H/HeOuJ mice (normal TLR4), Ang-II increased the systolic and diastolic BP commencing one week after Ang-II pump insertion (Fig. 1A and B). In contrast, C3H/HeJ mice (TLR4 deficiency) demonstrated blunted response to Ang-II; both systolic and diastolic BP increased after the second week and was significantly lower compared to the mice with normal TLR4 (Fig. 1A–C) at the end of 4-week period. High dose Ang-II increased renal cortical resistive index (Fig. 2A and B) and decreased blood flow (Fig. 3A and B, black arrow) in mice with normal TLR4 to a greater extent compared to TLR4 deficiency mice (Figs 2 and 3).

**Figure 4:**
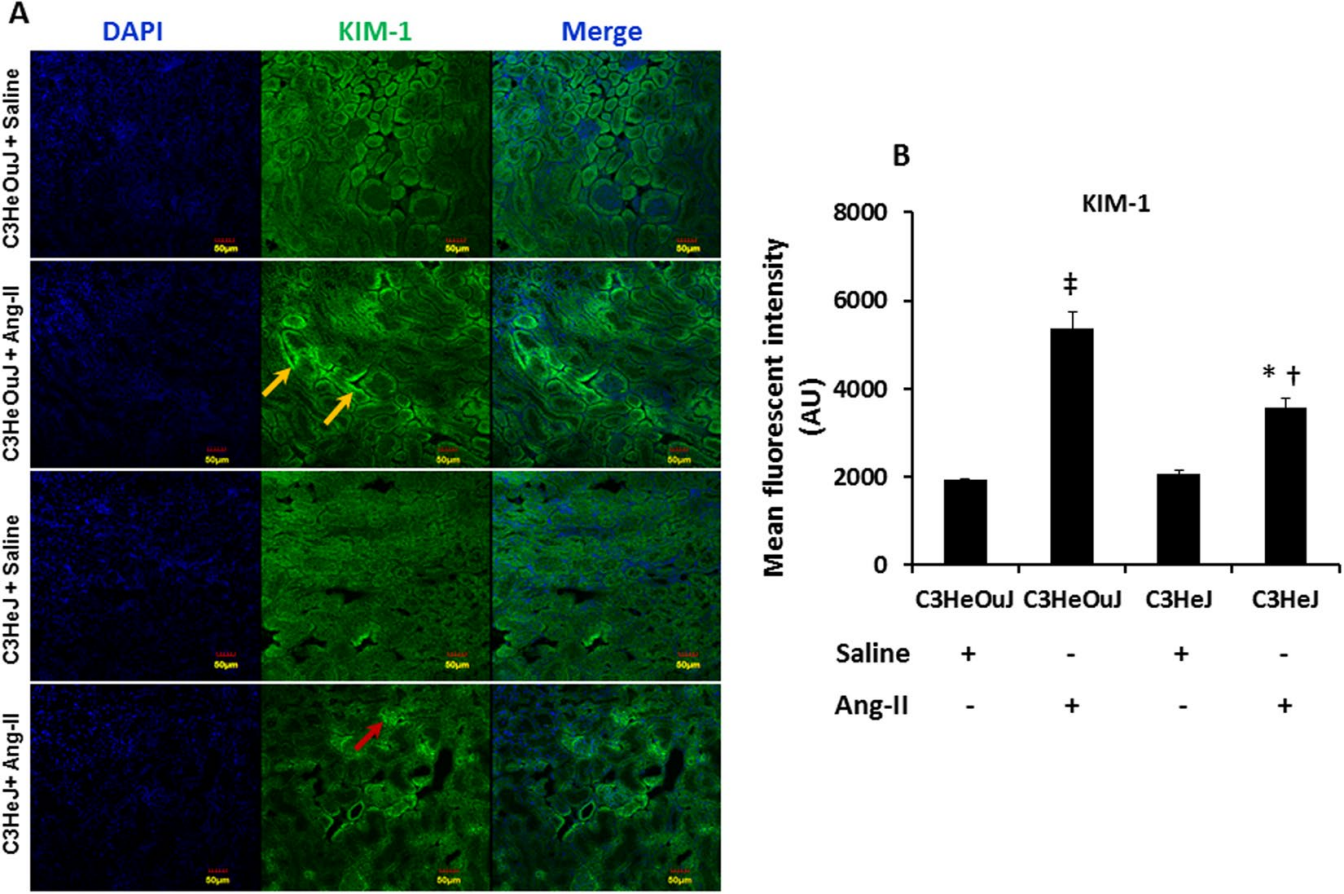
TLR4 deficiency protects the kidney from Ang-II mediated injury. (**A**) Representative images show Kidney injury molecule-1 immunofluorescence. Kidney sections from mice with normal TLR4 show increased KIM-1 fluorescence to Ang-II treatment in tubular areas (yellow arrows) compared to TLR4 deficiency mice (red arrow). (**B**) Data shows mean fluorescent intensity ± SEM. n = 5/group, tested by Kruskal-Wallis test. Scale bar - 50 µm. Magnification ×20. *p < 0.05 vs. C3HeJ + saline, ^†^p < 0.05 vs. C3HeOuJ + Ang-II, ^‡^p < 0.05 vs. C3HeOuJ + saline. C3HeOuJ: normal TLR4, C3HeJ: TLR4 deficiency.

Kidney injury molecule-1 (KIM-1) is a transmembrane glycoprotein expressed by the tubular cells following injury. We therefore evaluated the expression KIM-1 by immunostaining as an indicator of renal injury. In mice receiving saline treatment with normal TLR4 and TLR4 deficiency, there was no difference in the expression of KIM-1 (Fig. 4A and B). In response to Ang-II treatment, mice with normal TLR4 showed intense KIM-1 staining in the tubular areas (yellow arrows) in the renal medulla whereas, mice with TLR4 deficiency showed significant reduction in KIM-1 (red arrow, Fig. 4A and B).

### TLR4 deficiency diminishes Ang-II-induced ROS and NADPH oxidase 4

Reactive oxygen species (ROS) is implicated in hypertension induced renal damage. Since NADPH oxidase system is an important source of oxygen radicals and because the NADPH oxidase isoform 4 (Nox4) is highly expressed in the kidney, we quantified the levels of oxidative stress by dihydroethidium (DHE) staining and the protein and mRNA expression of Nox4 and its subunit, p22^PHOX^.

**Figure 5:**
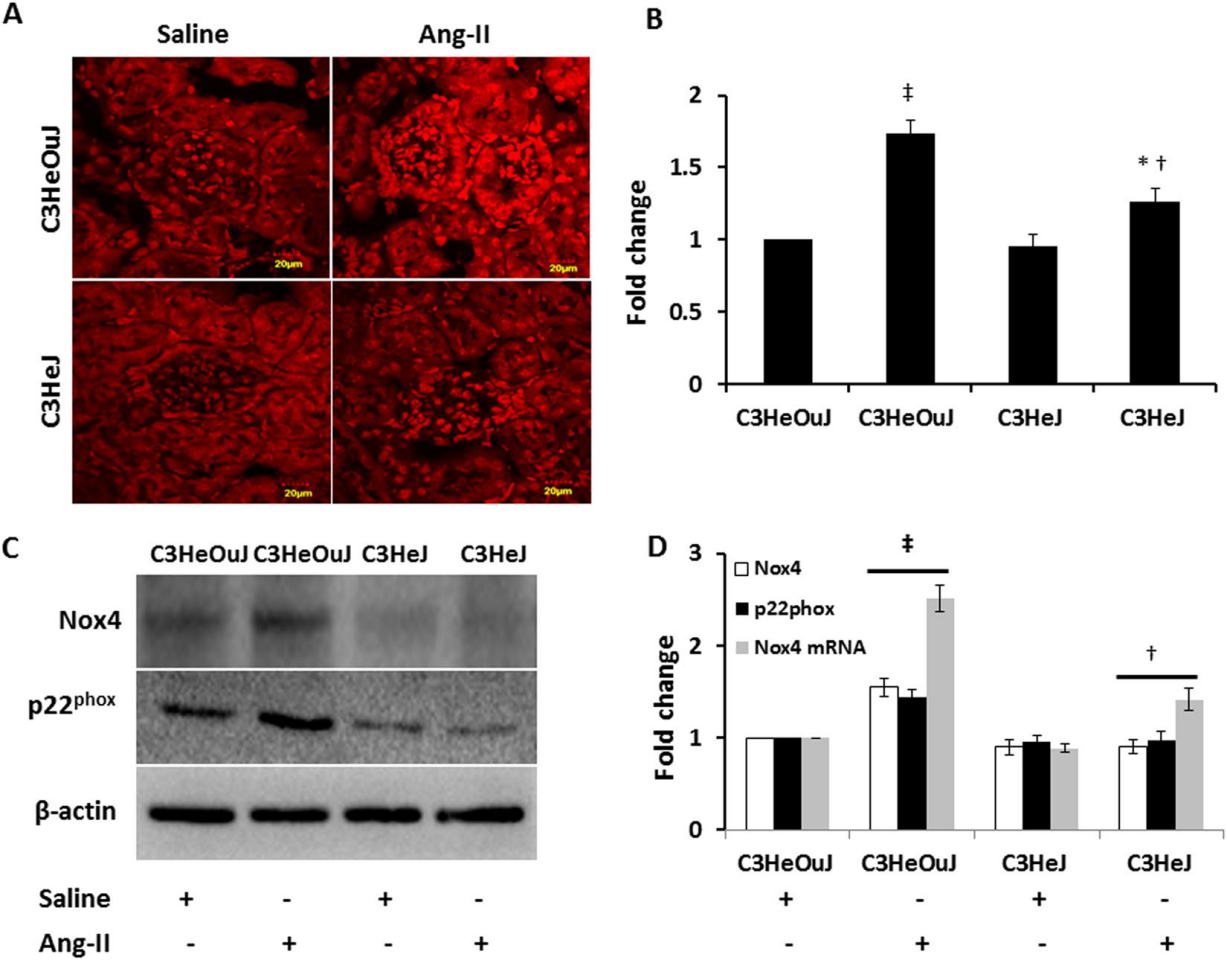
TLR4 deficiency reduces Ang-II-induced oxidative fluorescence and Nox4 and p22^PHOX^ expression. (**A**) Representative images of dihydroethidium (DHE) stained kidneys. Sections from mice with normal TLR4 exhibit intense DHE fluorescence suggesting increased oxidative stress. (**B**) Data shows fold change of mean intensity ± SEM. (**C**) Representative cropped immunoblot images of Nox4 and p22^PHOX^. Fifty micrograms of protein from each group were separated on SDS-PAGE and incubated with appropriate antibodies overnight. (**D**) mRNA fold change of Nox4 assessed by real-time PCR. Results are expressed as fold change relative to control mice (C3HeOuJ + Saline). Immunoblot data was normalized to β-actin. Values are presented as mean ± SEM. n = 6/group, tested by Kruskal-Wallis test and Mann-Whitney rank sum test. Scale bar: 20 µm. Magnification ×60. *p < 0.05 vs. C3HeJ + saline, ^†^p < 0.05 vs. C3HeOuJ + Ang-II, ^‡^p < 0.05 vs. C3HeOuJ + saline. C3HeOuJ: normal TLR4, C3HeJ: TLR4 deficiency.

In saline treated mice with normal TLR4 and TLR4 deficiency, there was no difference in the fluorescence intensity to DHE staining. In contrast, there was intense fluorescence to Ang-II treatment in mice with normal TLR4 (1.7-fold vs. saline control) which was predominantly seen in the tubular areas followed by glomeruli (Fig. 5A and B). In TLR4 deficient mice, there was 1.26-fold increase in fluorescence with Ang-II treatment compared to its saline treated control (Fig. 5A and B). The DHE fluorescence was significantly increased in Ang-II treated mice with normal TLR4 compared to mice with deficient TLR4 receiving similar treatment (Fig. 5A and B).

In saline treated animals with normal TLR4 and TLR4 deficiency, there was no difference in Nox4 and p22^phox^ expression (Fig. 5C and D). In response to Ang-II treatment, Nox4 expression was increased in mice with normal TLR4, while there was no change in mice with TLR4 deficiency (Fig. 5C and D). The mRNA levels of Nox4 reflected its protein expression in all the groups (Fig. 5D).

In mice with normal TLR4, Ang-II showed upregulation of p22^phox^ compared to its saline treated control and mice with TLR4 deficiency receiving Ang-II treatment (Fig. 5C and D). There was no change in the expression of p22^phox^ in mice with TLR4 deficiency treated with saline or Ang-II treatment (Fig. 5C and D).

### TLR4 deficient mice exhibit increased antioxidant defense mechanisms and maintain ATP production

**Figure 6:**
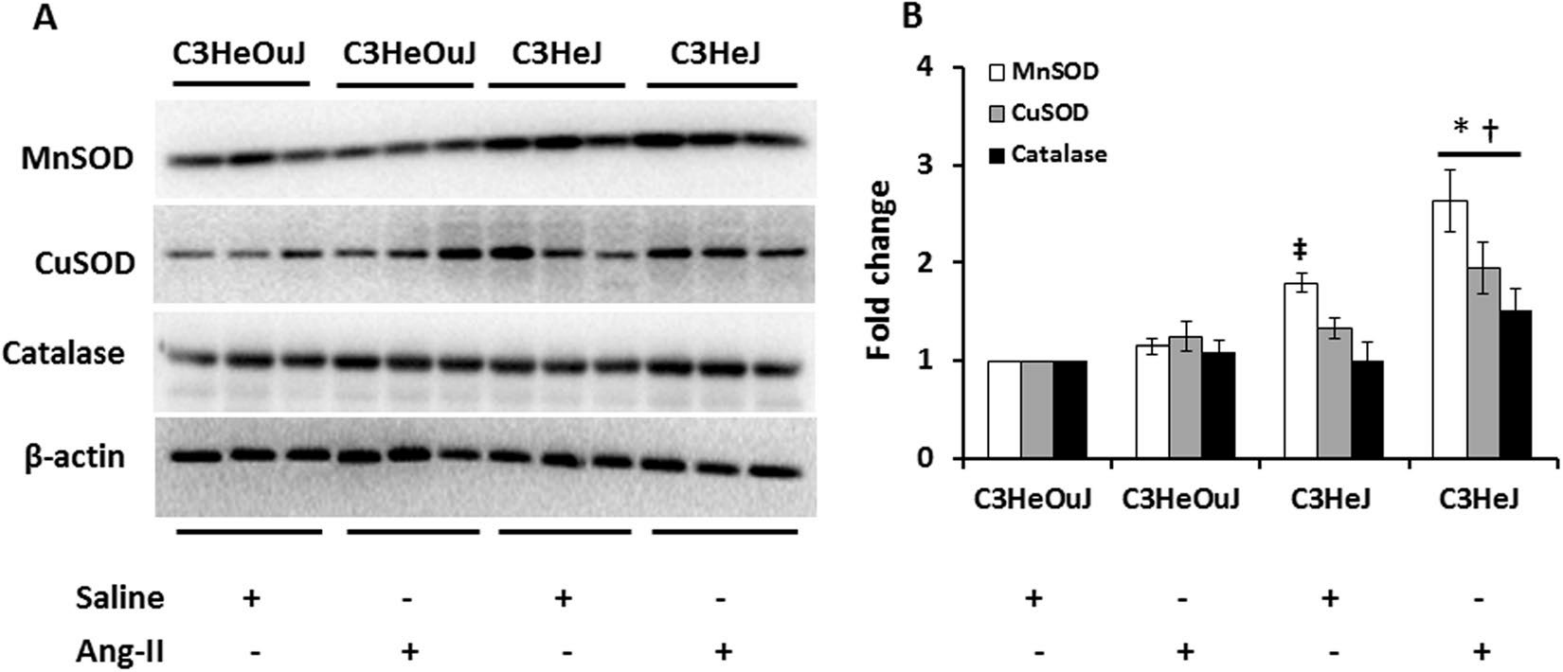
The expression of MnSOD, CuSOD and Catalase is upregulated in Ang-II treated TLR4 deficiency mice. (**A**) Fifty micrograms of protein from each group were separated on SDS-PAGE and incubated with appropriate antibodies overnight. (**B**) Data was normalized to β-actin and are presented as mean ± SEM. *p < 0.05 vs. C3H/HeJ + Saline, ^†^p < 0.05 vs. C3H/HeOuJ groups. n = 6/group.

Excess generation of superoxide radicals is an important cause of hypertension induced cellular injury. To determine whether the expression of antioxidant enzymes in mitochondria and cytosol are affected by Ang-II in TLR4 deficiency, the levels of manganese superoxide dismutase (MnSOD), copper superoxide dismutase (CuSOD) and catalase were quantified. In saline treatment groups, the basal expression of MnSOD was increased in TLR4 deficient mice compared to mice with normal TLR4 (Fig. 6A and B). In mice with normal TLR4, Ang-II treatment did not change the expression of MnSOD compared to its saline treated control. However, in mice with TLR4 deficiency, Ang-II treatment further enhanced the expression of mitochondrial MnSOD (Fig. 6A and B) compared to its saline control and mice with normal TLR4 receiving Ang-II treatment (Fig. 6A and B).

There was no change in the expression of CuSOD in mice with normal TLR4 compared to mice with TLR4 deficiency in response to saline treatment (Fig. 6A and B). In mice with normal TLR4, Ang-II treatment did not change the expression of CuSOD compared to its saline control. In contrast, Ang-II treatment significantly increased the expression of CuSOD in mice with TLR4 deficiency compared to its saline control and mice with normal TLR4 receiving Ang-II (Fig. 6A and B).

Hydrogen peroxide (H_2_O_2_) is a byproduct of mitochondrial respiration and the enzyme, catalase, degrades H_2_O_2_ to oxygen and water to maintain intracellular redox balance thus protecting the cells from ROS-induced injury. The expression of catalase remained similar in saline treated groups with normal TLR4 and TLR4 deficiency (Fig. 6A and B). In mice with normal TLR4, there was no difference in the catalase expression between saline and Ang-II treatment (Fig. 6A and B). In contrast, catalase expression was upregulated in mice with TLR4 deficiency in response to Ang-II compared to its saline control and Ang-II treated mice with normal TLR4 (Fig. 6A and B).

**Figure 7:**
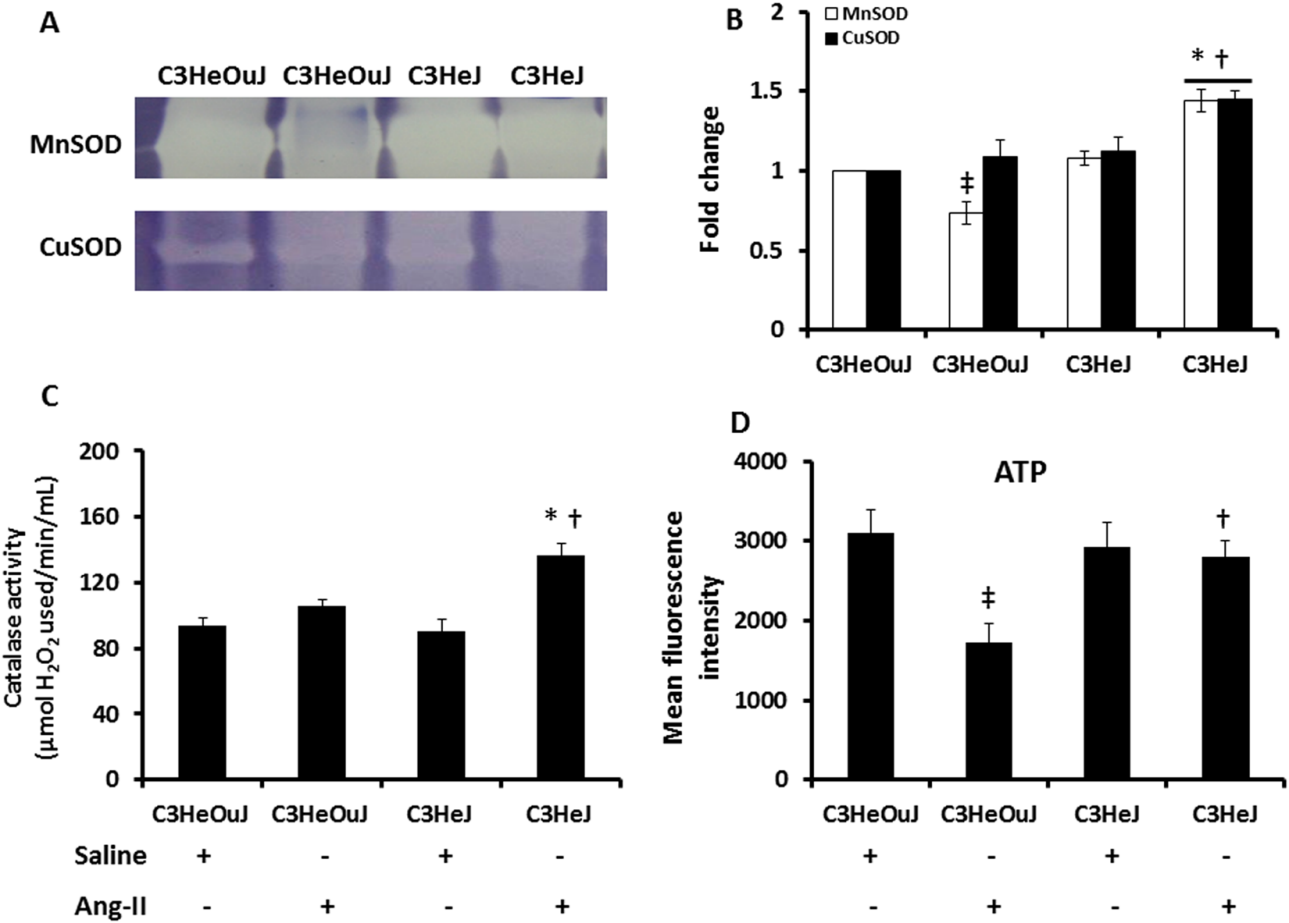
The enzyme activity of MnSOD, CuSOD, catalase and the levels of ATP are decreased following Ang-II treatment in mice with normal TLR4. (**A**) Representative images of gels stained for MnSOD and CuSOD activity. Hundred micrograms of protein were separated in 12% native gels as described in the Materials and Methods. (**B**) Data shows fold change of mean intensity ± SEM, n = 6/group. (**C**) The residual amount of H_2_O_2_ was used as a surrogate for catalase activity. Data shows catalase activity (µmol of H_2_O_2_ used/min/mL) as mean ± SEM. (**D**) Data showing ATP levels in all groups as mean fluorescent intensity ± SEM. n = 5/group, tested by Kruskal-Wallis test and Mann-Whitney rank sum test. *p < 0.05 vs. C3HeJ + saline, ^†^p < 0.05 vs. C3HeOuJ + Ang-II, ^‡^p < 0.05 vs. C3HeOuJ + saline. C3HeOuJ: normal TLR4, C3HeJ: TLR4 deficiency.

In order to determine whether a change in the expression of MnSOD, CuSOD and catalase was associated with change in the enzyme activity, gel activity assay and residual H_2_O_2_ was measured. The enzyme activity observed in mice with normal TLR4 was considered as control for comparison. In saline treatment mice with normal TLR4 and TLR4 deficiency, the MnSOD activity remained similar (Fig. 7A and B). The MnSOD activity was lower in response to Ang-II treatment in mice with normal TLR4 compared to its saline control (Fig. 7A and B). In mice with TLR4 deficiency receiving Ang-II treatment, MnSOD activity was increased compared to its saline control and mice with normal TLR4 receiving Ang-II treatment (Fig. 7A and B).

There was no difference in CuSOD activity in mice with normal TLR4 and TLR4 deficiency receiving saline treatment. In mice with normal TLR4, CuSOD activity was unaffected by Ang-II treatment (Fig. 7A and B). In contrast, CuSOD activity was increased in mice with TLR4 deficiency compared to its saline control and Ang-II treated mice with normal TLR4 (Fig. 7A and B).

The amount of residual H_2_O_2_ is inversely proportional to the catalase activity. There was no difference in the catalase activity in the saline treatment groups (Fig. 7C). There was no change in the catalase activity following Ang-II treatment in mice with normal TLR4 suggesting impaired ability to breakdown of H_2_O_2_ and thus increased oxidative stress. In contrast, Ang-II treated TLR4 deficient mice showed increased catalase activity compared to mice with normal TLR4 receiving similar treatment and its respective saline treated control (Fig. 7C).

Since mitochondria is the main source of ATP generation in the cells and oxidative stress is known to impair ATP generation^21, 22^, we measured intracellular ATP as an indicator of mitochondrial function. The mean fluorescence was similar in saline treatment groups. In mice with normal TLR4, Ang-II treatment decreased the fluorescence compared to its respective saline treatment group (Fig. 7D). In contrast, in mice with TLR4 deficiency, there was no difference in ATP fluorescence to Ang-II treatment compared to its saline control suggesting better maintenance of mitochondrial function than mice with normal TLR4 receiving Ang-II (Fig. 7D).

### Macrophage mediated inflammation is mitigated by TLR4 deficiency

To determine whether TLR4 deficiency affects inflammatory response to Ang-II in the kidney, we examined the protein and mRNA expression of MCP-1 and MIP-2, and protein expression of TNF α and IL-6. Further, we investigated whether the production of the chemokines and cytokines was associated with classical activation of macrophages by using CD68 as a marker for pro-inflammatory M1 phenotype.

**Figure 8:**
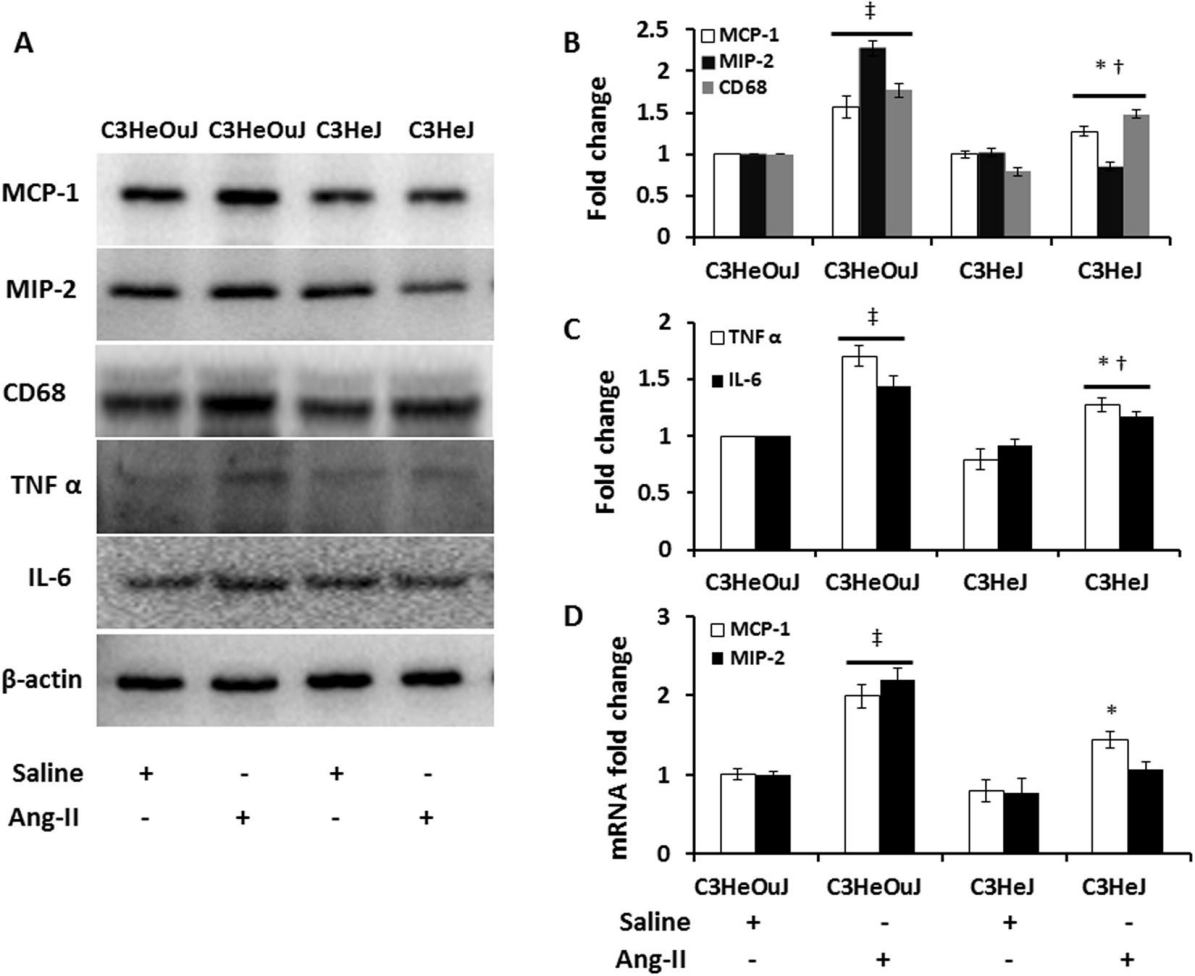
TLR4 deficiency reduces pro-inflammatory chemokines and cytokines expression and classically activated M1 macrophage. Fifty micrograms of protein from each group were separated on SDS-PAGE and incubated with appropriate antibodies overnight. (**A**) Representative cropped immunoblot images for MCP-1, MIP-2, CD68, a marker for M1 type macrophage, TNF α and IL-6 showing reduced expression in TLR4 deficiency mice in response to Ang-II treatment, (**B**,**C**) Data normalized to to β-actin and presented as mean ± SEM, (**D**) mRNA fold change for MCP-1 and MIP-2 assessed by real-time PCR. Results are expressed as fold change relative to control mice (C3HeOuJ + Saline). n = 5/group, tested by Kruskal-Wallis test and Mann-Whitney rank sum test. *p < 0.05 vs. C3HeJ + saline, ^†^p < 0.05 vs. C3HeOuJ + Ang-II, ^‡^p < 0.05 vs. C3HeOuJ + saline. C3HeOuJ: normal TLR4, C3HeJ: TLR4 deficiency.

In the saline treated mice with normal TLR4 or TLR4 deficiency, there was no difference in the protein expression of MCP-1, MIP-2 and CD68 (Fig. 8A and B). Ang-II treatment increased the expression of all three markers in mice with normal TLR4 compared to its saline treated control and mice with TLR4 deficiency receiving Ang-II treatment (Fig. 8A and B). In mice with TLR4 deficiency, the expression of MCP-1 and CD68 was increased and MIP-2 was decreased compared to its respective saline control (Fig. 8A and B). The expression of MCP-1, MIP-2 and CD68 was decreased in mice with TLR4 deficiency compared to mice with normal TLR4 receiving Ang-II treatment. The expression of inflammatory cytokines, TNF α and IL-6, did not differ between saline treated mice with or without normal TLR4. Both markers were increased in response to Ang-II treatment in mice with normal TLR4 compared to its saline control and mice with TLR4 deficiency receiving Ang-II treatment (Fig. 8A and C).

The mRNA levels of MCP-1 were increased in Ang-II treated mice with normal TLR4 compared to its saline treated control. Further, this level was higher compared to Ang-II treated mice with TLR4 deficiency (Fig. 8D). The level of MIP-2 was increased in mice with normal TLR4 to Ang-II treatment but remained unaffected in mice with TLR4 deficiency (Fig. 8D).

### TLR4 deficiency suppresses accumulation of bone marrow-derived fibroblasts and renal fibrosis

**Figure 9:**
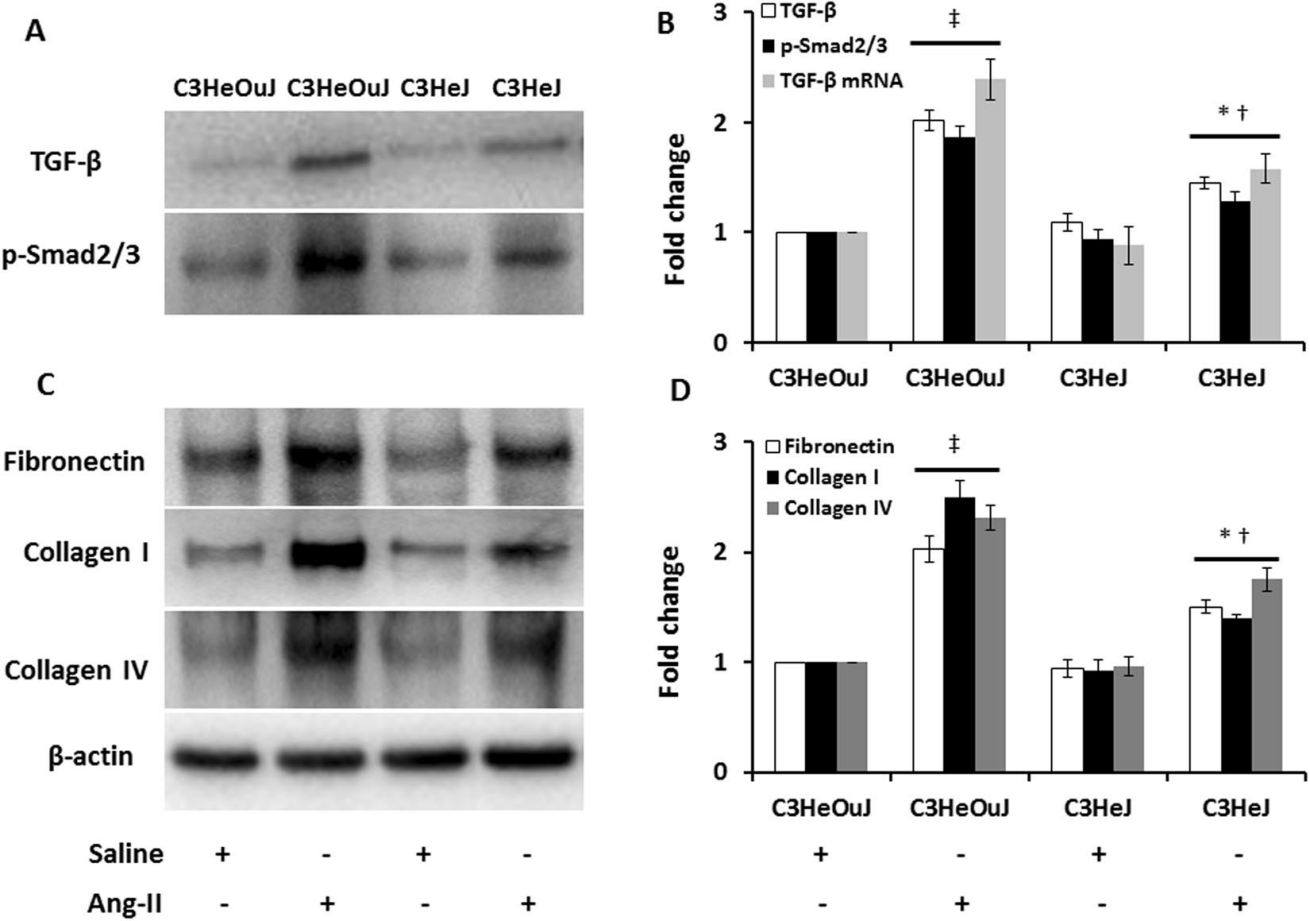
TLR4 deficiency decreases the expression and level of TGF-β, p-Smad2/3 and extracellular matrix proteins in Ang-II treated mice. (**A**) Representative cropped immunoblot images for TGF-β, p-Smad2/3, (**B**) mRNA fold change of TGF-β assessed by real-time PCR, (**C**) Representative cropped immunoblot images for fibronectin, collagen I and collagen IV, (**B**,**D**) Data was normalized to β-actin and presented as mean ± SEM. n = 6/group, tested by Kruskal-Wallis test and Mann-Whitney rank sum test. *p < 0.05 vs. C3HeJ + saline, ^†^p < 0.05 vs. C3HeOuJ + Ang-II, ^‡^p < 0.05 vs. C3HeOuJ + saline. C3HeOuJ: normal TLR4, C3HeJ: TLR4 deficiency.

Ang-II is known to upregulate TGF-β production, an important molecule implicated in the development of renal interstitial fibrosis^23, 24^. TGF-β causes renal fibrosis via activation of Smad2 and Smad3 leading to complex formation with Smad4^25^. Subsequent translocation of the complex into the nucleus targets the genes involved in extracellular matrix protein synthesis^25^. We therefore investigated whether TGF-β production and downstream signaling involving p-Smad2/3 was affected in TLR4 deficiency. In saline treated mice with or without normal TLR4, there was no difference in TGF-β and p-Smad2/3 expression (Fig. 9A and B). In mice with normal TLR4, Ang-II significantly increased the expression of TGF-β and p-Smad2/3 compared to its respective saline treated control. In mice with TLR4 deficiency, Ang-II treatment increased TGF-β and p-Smad2/3 expression compared to its saline treated control but the expression was lower than that observed in mice with normal TLR4 receiving Ang-II (Fig. 9A and B). The mRNA levels for TGF-β showed similar changes as protein expression (Fig. 9B).

Since hypertensive nephrosclerosis is characterized by glomerular and tubular changes, we investigated the deposition of excess ECM proteins, fibronectin, collagen I and collagen IV. There was no difference in the expression of the proteins in saline treated mice with or without normal TLR4 (Fig. 9C and D). All three ECM proteins above were significantly increased in mice with normal TLR4 in response to Ang-II treatment compared to saline treated control (Fig. 9C and D). Although the expression of fibronectin, collagen I and collagen IV were increased in mice with TLR4 deficiency receiving Ang-II, the levels were much lower than that observed in mice with normal TLR4 receiving Ang-II (Fig. 9C and D).

**Figure 10:**
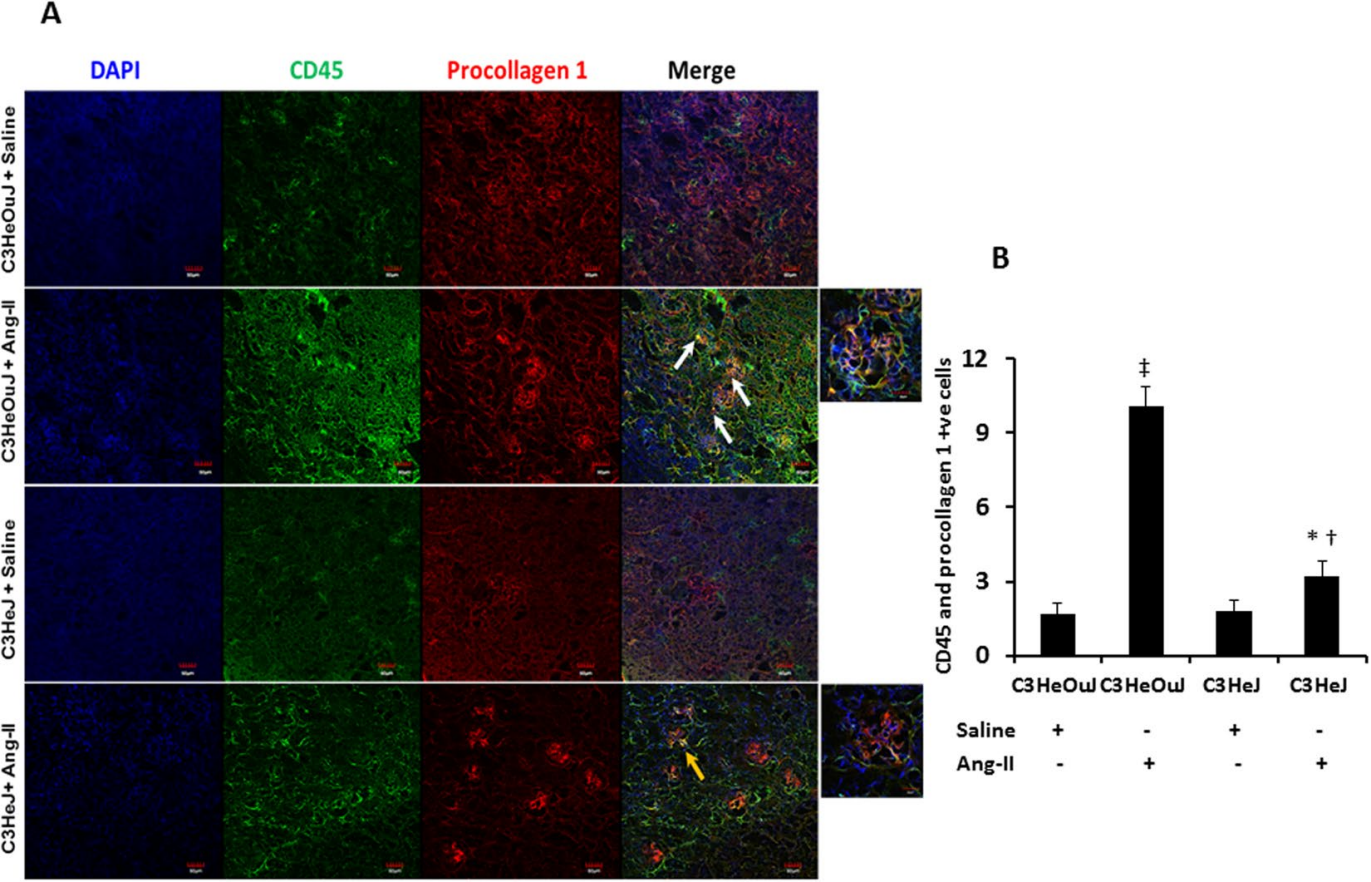
Infiltration of bone marrow derived fibroblasts is decreased in TLR4 deficiency mice following Ang-II treatment. (**A**) Representative images of kidneys stained for CD45 (green) and procollagen 1 (red) from C3H/HeOuJ and C3H/HeJ. C3HeOuJ mice treated with Ang-II show increased colocalization for both antigens (white arrows) compared to C3HeJ mice (yellow arrow). Outset images show yellow color in areas of colocalization. (**B**) Quantitative analysis of no. of cells positive for CD45 and procollagen 1. Data is presented as mean ± SEM. n = 5/group tested by Kruskal-Wallis test and Mann-Whitney rank sum test. Magnification ×20, Scale bar: 20 µm. Outset image magnification ×100. Scale bar: 20 µm. *p < 0.05 vs. C3HeJ + saline, ^†^p < 0.05 vs. C3HeOuJ + Ang-II, ^‡^p < 0.05 vs. C3HeOuJ + saline. C3HeOuJ: normal TLR4, C3HeJ: TLR4 deficiency.

TGF-β is involved in the recruitment and differentiation of bone marrow-derived fibroblasts which play an important role in renal pathogenesis and regeneration following kidney injury^26–29^. To determine whether TLR4 deficiency affects the infiltration of bone marrow-derived fibroblasts into the kidney, we quantified the expression of CD45, a marker expressed by cells of hematopoietic origin and procollagen 1, a mesenchymal marker. Our results showed that in mice with normal TLR4, Ang-II treatment increased the accumulation of CD45 and procollagen 1 positive cells (white arrows) compared to mice with TLR4 deficiency (yellow arrow, Fig. 10A and B).

**Figure 11:**
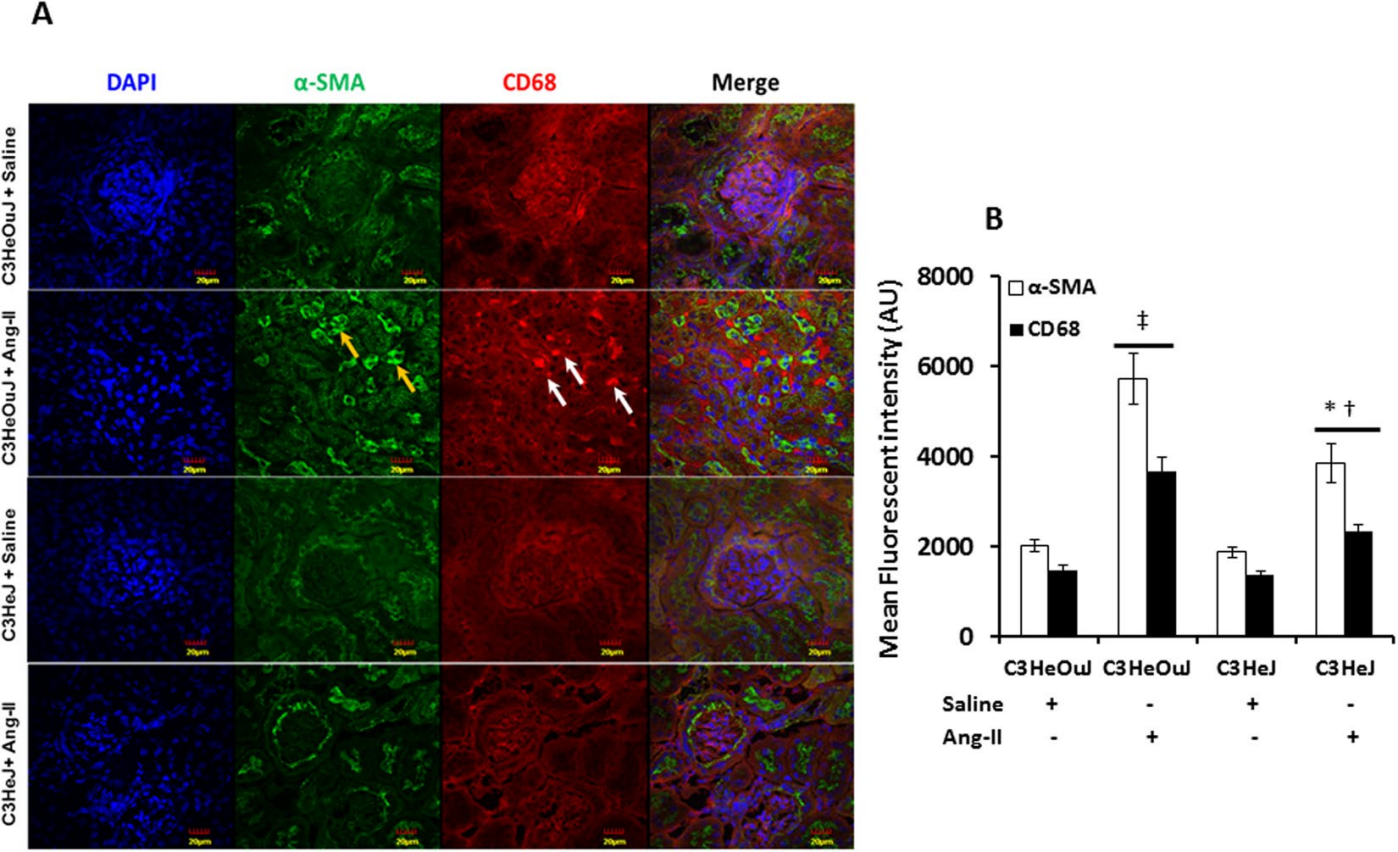
The expression of α-SMA and CD68 is decreased in mice with TLR4 deficiency. (**A**) Representative immunofluorescence image for fibroblast marker, α-SMA and CD68, a marker for inflammatory macrophage. The α-SMA (yellow arrows) and CD68 (white arrows) expression is increased in tubulo-interstitial areas in mice with normal TLR4 to Ang-II treatment. (**B**) Data is presented as mean fluorescent intensity ± SEM. n = 5/group, tested by Kruskal-Wallis test and Mann-Whitney rank sum test. Magnification ×60. Scale bar: 20 µm. *p < 0.05 vs. C3HeJ + saline, ^†^p < 0.05 vs. C3HeOuJ + Ang-II, ^‡^p < 0.05 vs. C3HeOuJ + saline. C3HeOuJ: normal TLR4, C3HeJ: TLR4 deficiency.

Myofibroblasts are associated with fibrosis in several models of renal injury and are known to express α-smooth muscle actin (α-SMA) abundantly^30^. We therefore examined the expression of α-SMA, indicative of cells responsible for extracellular matrix (ECM) protein accumulation. There was no change in the expression α-SMA in mice with or without normal TLR4 treated with saline (Fig. 11A and B). In contrast, mice with normal TLR4 receiving Ang-II treatment showed increased α-SMA in the tubular areas predominantly (yellow arrows, Fig. 11A) compared to saline treated respective control and Ang-II treated mice with TLR4 deficiency (Fig. 11A and B). Immunohistochemistry revealed increased expression for CD68, a marker for inflammatory macrophage in mice with normal TLR4 to Ang-II treatment compared to its saline control and Ang-II treated C3HeJ mice with TLR4 deficiency (Fig. 11A and B).

**Figure 12:**
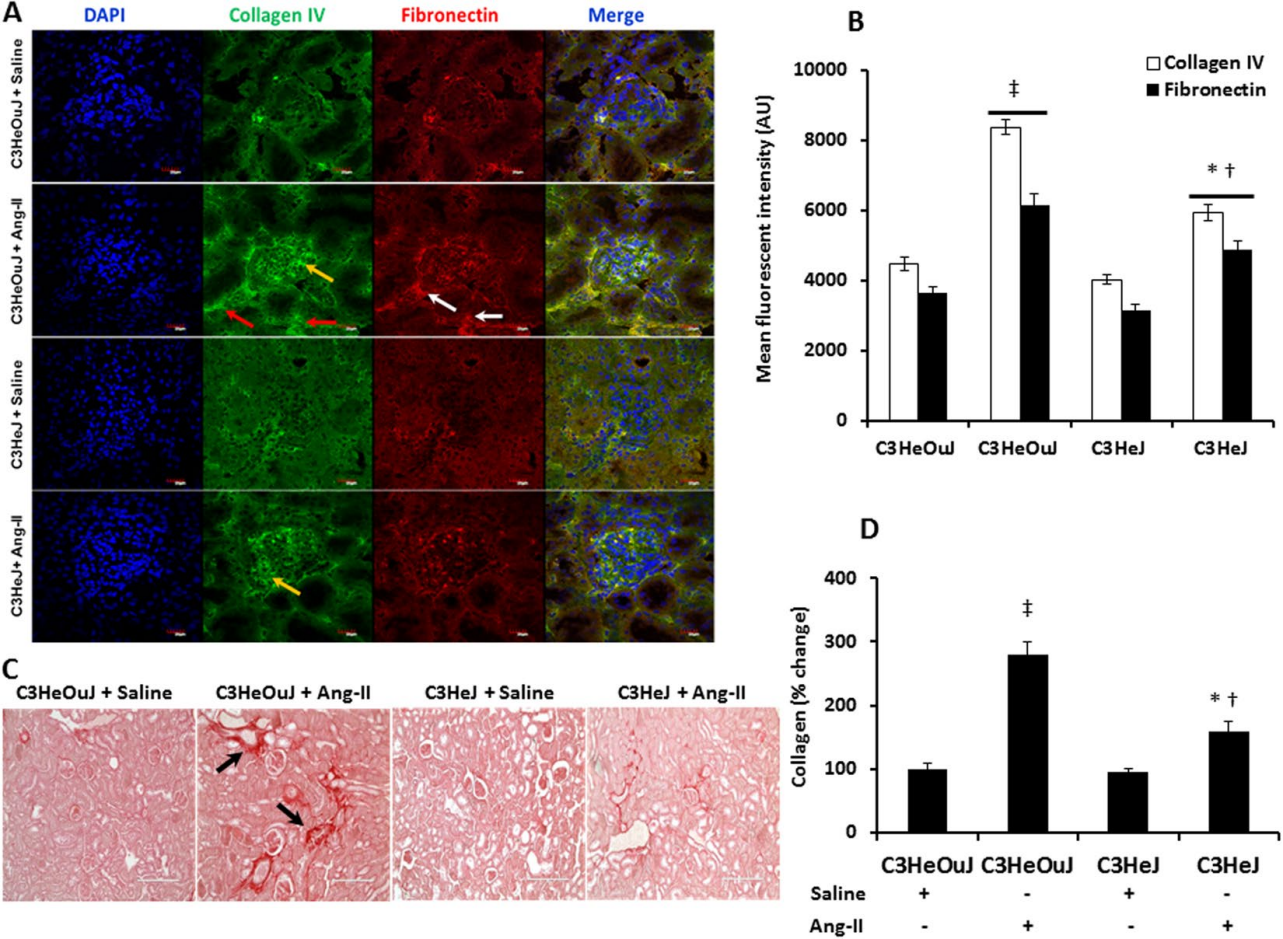
TLR4 deficiency decreases extracellular proteins, collagen and fibronectin deposition in the kidney. (**A**) Representative images of kidneys stained for collagen IV in the glomerular basement membrane (yellow arrow) and tubules (red arrows) and fibronectin (white arrows). (**B**) Quantitative analysis of fluorescent intensity, data is presented as mean ± SEM. n = 5/group, tested by Kruskal-Wallis test and Mann-Whitney rank sum test. Magnification ×60. Scale bar: 20 µm. (**C**) Representative images for Picrosirius red staining for collagen (Black arrows). (**D**) Data is presented as mean percent change from control mice ± SEM. n = 5/group, tested by Kruskal-Wallis test and Mann-Whitney rank sum test. Magnification ×20. Scale bar: 200 µm. n = 6/group tested by Kruskal-Wallis test and Mann-Whitney rank sum test. *p < 0.05 vs. C3HeJ + saline, ^†^p < 0.05 vs. C3HeOuJ + Ang-II, ^‡^p < 0.05 vs. C3HeOuJ + saline. C3HeOuJ: normal TLR4, C3HeJ: TLR4 deficiency.

In mice with normal TLR4 receiving Ang-II, immunostaining for collagen IV and fibronectin revealed excess deposition in both glomerular basement membrane and tubular areas (yellow and white arrows respectively, Fig. 12A and B) compared to mice with TLR4 deficiency receiving similar treatment. There was no change in the expression of both molecules in saline treated groups with or without normal TLR4 (Fig. 12A and B). The excess deposition of collagen was further confirmed with Picrosirius red stain which showed a marked increase in the renal cortex and juxtamedullary areas in mice with normal TLR4 treated with Ang-II compared to mice with TLR4 deficiency receiving Ang-II (Fig. 12C and D). There was no difference in picrosirius red stain in the saline treated groups with or without normal TLR4.

## Discussion

Oxidative stress and inflammation contribute to the development and progression of hypertension induced kidney damage; however, the relationship between the two pathological processes is incompletely understood. Recent studies suggest that TLRs particularly TLR4 signaling may mediate both oxidative stress and inflammation^11, 31^. We therefore chose C3H/HeJ^*Lps-d*^ mice representing TLR4 deficiency and C3H/HeOuJ with normal TLR4 to study the role of TLR4 in Ang-II-induced hypertension on renal injury and remodeling. C3H/HeJ^*Lps-d*^ mice have a mutation in the toll-like receptor 4 gene which renders it incapable of activating NF-_K_B^32^.

In this study, TLR4 deficiency mice showed blunted response to Ang-II induced hypertension compared to mice with normal TLR4. In addition, measurement of vascular indices revealed decreased intra-renal vascular resistance and increased renal cortical blood flow in mice with TLR4 deficiency compared to mice with normal TLR4. The cellular anti-oxidant mechanism was augmented in TLR4 deficient mice thereby reducing oxidative stress and intracellular ATP generation was better maintained. Further, TLR4 deficiency suppressed the expression of pro-inflammatory chemokines and cytokines (MCP-1, MIP2, and TNF α, IL-6) which was associated with decreased accumulation of M1 inflammatory macrophage. The expression of TGF-β was decreased including the infiltration of bone marrow-derived fibroblasts in TLR4 deficiency and was associated with reduced deposition of extracellular matrix proteins, such as, collagen I, collagen IV and fibronectin suggesting decreased fibrosis.

In earlier studies, TLR4 deficiency mice were found to have significantly lower systolic BP compared to other strains suggesting that genomic differences could display phenotypic variations^33, 34^. Anti-TLR4 antibody treatment in WT mice following Ang-II and in spontaneously hypertensive rats were shown to abrogate systolic, diastolic and mean BP increase^15, 35^. The results from the present study confirm the above findings and suggest an important role for TLR4 in hypertension.

In response to pathological stimuli, blood vessels undergo remodeling. Using a model of carotid artery ligation, Harmon *et al*. demonstrated that arteries from C3H/HeJ mice (TLR4 deficiency) were refractory to remodeling^36^. Interestingly, the first change seen in hypertension-induced target organ damage is small artery remodeling which involves increased intimal thickening and lumen narrowing resulting in increased peripheral vascular resistance. This is reflected as increased renal resistive index (RI) on ultrasonography as seen in the present study in mice with normal TLR4. An increase in the vascular resistance implies altered hemodynamics and changes to renal blood flow. In the present study, increased RI correlated with reduced cortical blood flow in mice with normal TLR4 compared to TLR4 deficiency mice. Taken together, these results suggest that TLR4 deficiency protects the renal vasculature from Ang-II induced remodeling whereas mice with normal TLR4 develop vascular dysfunction suggestive of arteriosclerosis in the kidney.

The nicotinamide adenine dinucleotide phosphate (NADPH) family is one of the major sources of ROS generation in the body. The isoform, NADPH oxidase 4 (Nox4) is highly expressed in the kidney and its activity is dependent on the membrane subunit, p22^phox^. TLR4 has the ability to bind directly to Nox4 to generate ROS in response to LPS stimulation and inhibition of TLR4 signaling was found to attenuate oxidative stress in the diabetic kidney^37, 38^. Our results support these earlier findings. In addition, we found that in the presence of TLR4, Ang-II upregulates Nox4 leading to ROS generation in the hypertensive kidney.

In the aerobic cells, the respiratory chain produces ROS continuously as a normal process. The superoxide (O_2_^−•^) generated is effectively scavenged by MnSOD in the mitochondrial matrix and CuSOD located in the cytosol and nucleus to produce H_2_O_2_. Subsequently, the enzymes catalase and glutathione peroxidase metabolize H_2_O_2_ to oxygen and water. A deficiency of the anti-oxidant mechanism above is detrimental to the tissues. In this study, mice with TLR4 deficiency exhibited upregulation of MnSOD, CuSOD and catalase activities in response to Ang-II infusion suggesting a robust anti-oxidant mechanism. Inability to mount an effective defense against oxidative stress has been demonstrated in spontaneously hypertensive rat (SHR) kidney and aorta^39, 40^. Indeed, discordance between the expression of MnSOD, CuSOD and catalase proteins and their activity is a major cause of oxidative stress in SHR kidney leading to damage^39^.

Ang-II is a potent stimulator of mitochondrial ROS production and studies have shown that Ang-II can increase state 4 and decrease state 3 respirations which can be associated with decreased ATP synthesis^22^. Further, Ang-II infusion has also been shown to reduce the expression of genes involved in electron transport chain and Krebs cycle that can adversely affect energy metabolism^41^. Also, there is supporting evidence suggesting that TLR4 mediated mitochondrial damage can suppress ATP production in Kupffer cells and loss of immune function following hemorrhagic shock whereas, TLR4 mutant mice maintained normal ATP levels and improved immune function^42^. In the present study, our results showing impaired ATP synthesis following Ang-II infusion in mice with normal TLR4 is in agreement with the earlier studies above.

During hypertension, ROS can trigger the activation of transcription factors of genes that encode for chemokines and cytokines thereby promoting the accumulation of inflammatory cells^1, 43, 44^. The induction of chemokines, MCP-1, MIP-2 has been demonstrated in experimental hypertensive kidney models and human studies^45–47^. Subsequent recruitment of inflammatory cells and production of cytokines contribute to the progression of kidney disease. Macrophage mediated inflammation has been demonstrated in hypertension induced kidney damage^7^ and its potential pathway may involve TLR4 activation^48^. TLR4 is expressed by macrophages and by the cells in the kidney such as, tubular epithelium, mesangial cells and podocytes^49, 50^ and the latter cells show further upregulation of TLR4 to Ang-II treatment^15, 51, 52^. Inhibition of TLR4 has been shown to reduce BP and inflammation in the mesenteric arteries of SHRs^48^. TLR4 mediated activation of macrophages has been shown to contribute to an inflammatory environment in atherosclerotic plaque formation^53^. In the present study, our results show that, in the absence of TLR4, chemokines (MCP-1, MIP-2) and cytokines (TNF α, IL-6) production are attenuated in response to Ang-II treatment. An earlier study demonstrated that in response to lipopolysaccharide treatment, macrophage activation was impaired in CH3/HeJ^*Lps-d*^ strain (TLR4 deficiency) compared to macrophages from BDF-1 mice^54^. The macrophages from TLR4 deficiency mice required additional stimuli with silica for activation and secretion of IL-1^54^. This could explain the decreased expression of M1 macrophage marker, CD68 in response to Ang-II in the present study.

Recently, bone marrow-derived fibroblasts were shown to contribute to renal fibrosis in models of unilateral ureteral obstruction and ischemia reperfusion injury^26, 55, 56^. Further, under the influence of TGF-β, the bone marrow-derived precursors were shown to differentiate into myofibroblasts leading to excessive deposition of extracellular matrix proteins^55, 56^. It is well-known that Ang-II is a potent inducer of TGF-β and its subsequent downstream signaling pathways involving Smad2 and Smad 3^23^. In the kidney, TGF-β is produced predominantly by tubular epithelial and mesangial cells in response to Ang-II^57^. Further Ang-II can stimulate other cell types such as mesenchymal cells, myofibroblasts and inflammatory cells to produce TGF-β. In a study by Pulskens *et al*., TLR4 activation by TGF-β increased collagen synthesis in both renal tubular epithelial cells and myofibroblasts suggesting significant contribution from both cell types^16^. In the present study, increased expression of CD45 and procollagen 1 +ve cells, and α-SMA suggests significant production of TGF-β by myofibroblasts.

The absence of chemokine, CXCL16, was recently shown to attenuate Ang-II induced TGF-β1 expression in mice kidney and thus fibrosis^58^. In the present study, we show that TLR4 deficiency suppresses the recruitment of bone marrow-derived fibroblasts in the kidney. In addition, decreased expression of α-SMA positive cells suggests reduced myofibroblast accumulation in the kidney. These changes were associated with decrease in the accumulation of ECM proteins collagen I, collagen IV and fibronectin.

In summary, our study demonstrates that TLR4 deficiency mice are protected from Ang-II induced renal injury by a robust antioxidant mechanism in the cells. The decrease in the oxidative stress is associated with attenuation of pro-inflammatory chemokine and cytokine production and macrophage activation. Further, we show that TLR4 may play a role in the recruitment of bone marrow-derived fibroblasts into the kidney via TGF-β activation and TLR4 deficiency suppresses their accumulation to decrease renal fibrosis. Additional studies are required to identify TLR4 mediated signaling mechanisms involved in ROS generation and macrophage activation and delineate the crosstalk between oxidative stress and renal inflammation to identify potential therapeutic targets to reduce hypertension induced renal damage.

## Materials and Methods

All animal protocols were performed in accordance with institutional animal care guidelines and conform to the *Guide for the Care and Use of Laboratory Animals* published by the U.S. National Institutes of Health (NIH Publication, 2011). This study was approved by Institutional Animal Care and Use Committee (IACUC) of the University of Louisville School of Medicine. C3H/HeJ (Tlr4^*Lps-d*^, Stock no.: 000659) and C3H/HeOuJ (Stock no.: 000635) mice aged 10–12 weeks were purchased from Jackson Laboratory (Bar Harbor, ME). The C3H/HeJ strain has dysfunctional TLR4 and is therefore TLR4 deficient whereas, the C3H/HeOuJ is a sub strain of C3H/HeJ and has normal TLR4. The animals were fed standard chow and tap water ad libitum. The animals were treated without or with angiotensin-II (1000 ng/kg/min) for 4-week period. Blood pressure (BP) was measured by tail cuff method at 0, 1, 2, 3 and 4 weeks using Coda™ non-invasive BP system (Kent Scientific Corporation, Torrington, CT) as described before^59^.

Antibodies to Nox4, p22^phox^, CuSOD, catalase, MIP-2, TNF α, IL-6, Collagen IV, CD45, Procollagen 1, β-Actin, and GAPDH were from Santa Cruz Biotechnology (Dallas, TX), MnSOD and Phospho-Smad2/3 was from Cell Signaling (Danvers, MA), αSMA, CD68, KIM-1, MCP-1 and Fibronectin from Abcam (Cambridge, MA), TGF-β from Millipore (Billerica MA), anti-mouse alexa fluor 488 and alexa flour 594 were purchased from Invitrogen (Carlsbad, CA). All chemicals in activity assays were purchased from Sigma Aldrich (St. Louis, MO).

### Renal ultrasound and cortical blood flow measurement

Ultrasonography was performed to measure the blood flow in the renal cortex as described before^59^. Briefly, the animals were anesthetized by isoflurane inhalation and placed supine on a heated table. Body temperature was maintained at 37.5 °C. After depilation, acoustic gel (Other-Sonic; Pharmaceutial Innovations, Newark, NJ) was applied on the skin and imaging was performed using Vevo 2100 system (VisualSonics, Toronto, ON, Canada). The transducer, MS550D (22–55 MHz), was held immobile by an integrated rail system during imaging. The cortical vessels in the kidney were scanned in the short axis. All measurements were done on the left side and included peak systolic and end-diastolic blood flow velocity (mm/sec) in the Pulsed-Wave Doppler mode. Cine loops were exported and analyzed to obtain resistive index (RI).

Renal cortical blood flow was measured using Speckle Contrast Imager (Moor FLPI, Wilmington, DE) as described before^60^. Briefly, the left kidney was exposed through a dorsal incision and the camera was placed 15 cm. from the kidney. A live image flux was obtained with the camera set for low-resolution and high-speed. Line tracings from aorta, renal artery, renal vein and the renal cortex was recorded.

### Measurement of reactive oxygen species

To detect superoxide anion, freshly cut kidney sections were incubated with 5 µmol/L dihydroethidium (DHE) at 37 °C for 30 min in a humidified dark chamber. The presence of superoxide radicals results in oxidation of non-fluorescent hydroethidine to fluorescent 2-hydroxyethidium which intercalates with DNA in the nuclei. Images were captured with Olympus FluoView1000 (B&B Microscope Ltd., PA) at excitation/emission set at 510/595 nm.

### Immunoblot analysis

Whole kidney homogenates were separated on SDS-PAGE and transferred to Polyvinylidine difluoride (PVDF) membrane. The membranes were incubated with appropriate primary antibodies overnight and corresponding secondary antibody for 2 h. at room temperature. The immunoreactive bands were developed with chemiluminescence and visualized using ChemiDoc MP system (BioRad). Band intensities were quantified using ImageJ software (https://imagej.nih.gov/ij/). Full blots are presented in the supplementary file.

### Measurement of superoxide dismutase activity

Gel assay was used to determine the activity of superoxide dismutase-II (SOD-II) and catalase. Kidneys (25 mg) from all groups were homogenized in phosphate buffer (0.05 M, KH_2_PO4 and K_2_HPO4, pH 7.8) on ice. MnSOD and CuSOD activity assay was done using the technique described by Weydert *et al*.^61^. Briefly, 12% native gels were prepared and pre-electrophoresed for an hour at 40 mA at 4 °C and the gels were left in the cold room overnight. The following day, protein samples (100 µg) were loaded and run at 4 °C for 3 h in pre-electrophoresis buffer. The buffer was discarded and gels were run for further 2 h in fresh electrophoresis buffer at the same settings as before. The gels were stained with solution containing nitro blue tetrazolium (2.43 mM), 0.14 M riboflavin-5′-phosphate and 1.44 mg NaCN in 40 mL phosphate buffer for 40 min. at room temperature. The gels were washed with water and left overnight under room light. The gels were allowed to sit in water for another 12 h. without light and images were captured using digital camera.

### Catalase activity

The rate of degradation of H_2_O_2_ and thus the residual amount was used as a measure of catalase activity in the kidneys. The protocol used was described by Shangari and O’Brien^62^. Briefly, 30 mg of tissue was homogenized in homogenization buffer (Sucrose, 125 mM; mannitol, 125 mM; EGTA, 1 mM; HEPES, pH 7.2, 5 mM). Hundred microliters of 2.2 mM H_2_O_2_ was added to one mL of homogenate, mixed quickly and incubated at room temperature. A blank was prepared similarly using buffer instead of homogenate. At intervals of 0, 3, 5, and 10 min., 50 µL aliquots of blank, standards and samples were mixed with 950 µL of FOX 1 reagent containing ammonium ferrous sulfate (250 µM), xylenol orange (100 µM), sorbitol (0.1 M), H_2_SO_4_ (25 mM) and incubated for 30 min. at room temperature. The absorbance was read at 560 nm. The residual H_2_O_2_ and the catalase activity were calculated as described before^62^.

### ATP measurement

ATP in the kidney was measured using ATP assay kit (ab83355; Abcam, Cambridge, MA) following manufacturer’s instructions. Briefly, 10 mg of kidney was homogenized in 2 N ice cold perchloric acid and the supernatant was diluted with ATP assay buffer. The samples were neutralized and deproteinized with 2 M potassium hydroxide and loaded in duplicate into a microplate reader. ATP reaction mix and background control (50 µL) was added to the wells and incubated for 30 min. in dark. The fluorescence was read with the excitation/emission setting at 535/585 nm using SpectraMax M2e (Molecular Devices, Sunnyvale, CA). The mean fluorescent intensity was calculated relative to the standard curve.

### Immunohistochemistry and histology

Frozen kidney sections (5 µm) were air dried for 10 min. and fixed with 4% paraformaldehyde for 20 min. Following blocking (45 min.) at room temperature, sections were incubated with KIM-1, CD45, Procollagen A1, collagen IV, fibronectin, αSMA and CD68 antibodies at 4 °C overnight. Immune labeling was done with appropriate Alexa Fluor 488 and AF 594 conjugated secondary antibodies for 90 min. at room temperature. Images were captured by Olympus FluoView1000 (B&B Microscope Ltd., PA). Mean fluorescent intensity was quantified using ImageJ software (https://imagej.nih.gov/ij/) and presented as bar graphs.

Harvested kidneys were fixed in 10% phosphate buffered formalin (Fisher Scientific Co.) for 72 hours and embedded in paraffin. Sections were cut at 6 µm thickness and stained for collagen with Picrosirius red stain kit following manufacturer’s instructions (Polysciences, Warrington, CA). Images were captured by EVOS FL auto imaging system (Life Technologies, Grand Island, NY) and fibrotic areas were quantified using ImageJ software (https://imagej.nih.gov/ij/).

### Gene expression levels

Total RNA was extracted from the kidney using the TRIzol isolation method (Life Technologies, Carlsbad, CA), and cDNA was synthesized using Im-Prom-II^TM^ Reverse Transcription System (Madison, WI) following manufacturer’s protocol. All the primers were purchased from Invitrogen (Carlsbad, CA). The mRNA levels were quantified by real-time PCR (Lightcycler® 96 system, Roche Diagnostics Corporation, Indianapolis, IN) using specific primers for each molecule. The primer sequences used are listed in Table 1.

**Table 1:** Primers used in the study.

Type	Forward sequence (5′-3′)	Reverse sequence (5′-3′)
MCP-1	ACCACCTCAAGCACTTCTGTAG	TTAAGGCATCACAGTCCGAGTC
MIP-2	GGAAGCCTGGATCGTACCTG	TGAAAGCCATCCGACTGCAT
Nox4	GTACAACCAAGGGCCAGAATAC	CAGTTGAGGTTCAGGACAGATG
TGF-β	CTATTGCTTCAGCTCCACAG	GACAGAAGTTGGCATGGTAG
GAPDH	GTCGTGGAGTCTACTGGTGT	TGCTGACAATCTTGAGTGAG

### Statistics

Statistical analysis was done using Primer of Biostatistics (7^th^ edition). Data is presented as mean ± SEM. The differences between groups were determined using ANOVA for parametric data and Kruskal-Wallis test for nonparametric data. The differences between two groups were determined by t-test/Mann-Whitney Rank Sum Test. A ‘p’ value < 0.05 was considered significant.

## Electronic supplementary material

Supplementary Information
