# Supplementary material for: Toll-like Receptor 4 Deficiency Reduces Oxidative Stress and Macrophage Mediated Inflammation in Hypertensive Kidney
Source: Sci Rep. 2017 Jul 25;7:6349. doi: 10.1038/s41598-017-06484-6 (PMC5526876; doi:10.1038/s41598-017-06484-6)
Supplement: Supplementary file 1 — Supplementary Information [file 41598_2017_6484_MOESM1_ESM.pdf]

Supplementary information

Toll-like Receptor 4 Deficiency Reduces Oxidative Stress and Macrophage Mediated Inflammation in Hypertensive Kidney

Sathnur Pushpakumar[1], MD, PhD, Lu Ren[1], PhD, Sourav Kundu[2], PhD, Alejandra Gamon[3], Suresh C. Tyagi[1], PhD, Utpal Sen[1], PhD*

[1]Department of Physiology, University of Louisville School of Medicine, Louisville, KY-40202

[2]Institute of Advanced Study in Science and Technology, Guwahati, Assam – 781035, India

[3]University of California, San Diego, CA 92093

Running title: TLR4 induced oxidative stress and renal inflammation

Keywords: TLR4, hypertension, oxidative stress, macrophage, bone marrow-derived fibroblast

Word count: 5487

Number of Figures: 12

No. of tables: 1

Correspondence:

Utpal Sen, Ph.D.
500 S Preston St.
HSC-A, Room 802,
University of Louisville,
KY-40202,
Ph: 502-852-2030;
Fax: 502-852-6239;
Email: utpal.sen@louisville.edu

Figure 5C

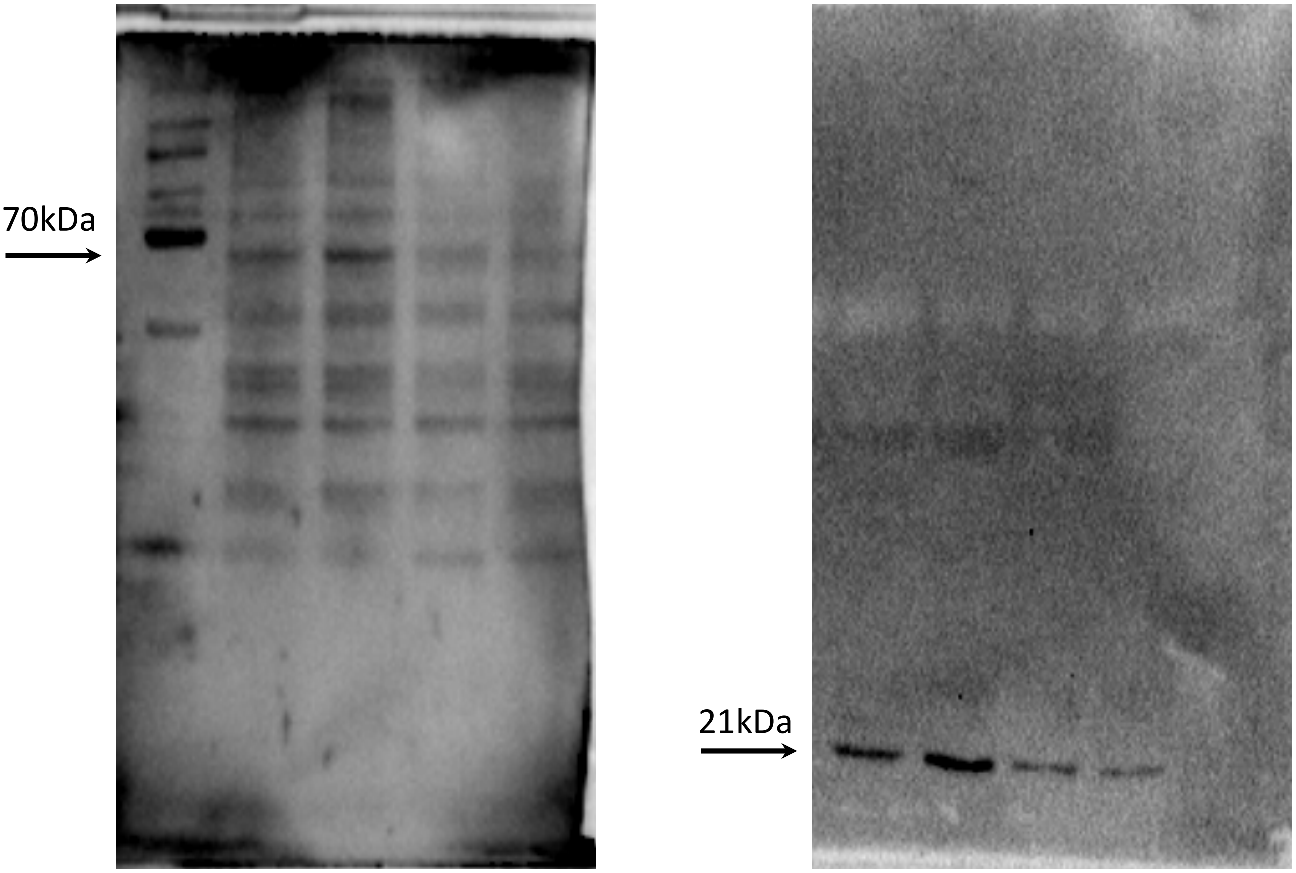

Nox4

p22-phox

Supplementary figure S1: Uncropped images for Nox4 and p22-phox.

Figure 8A

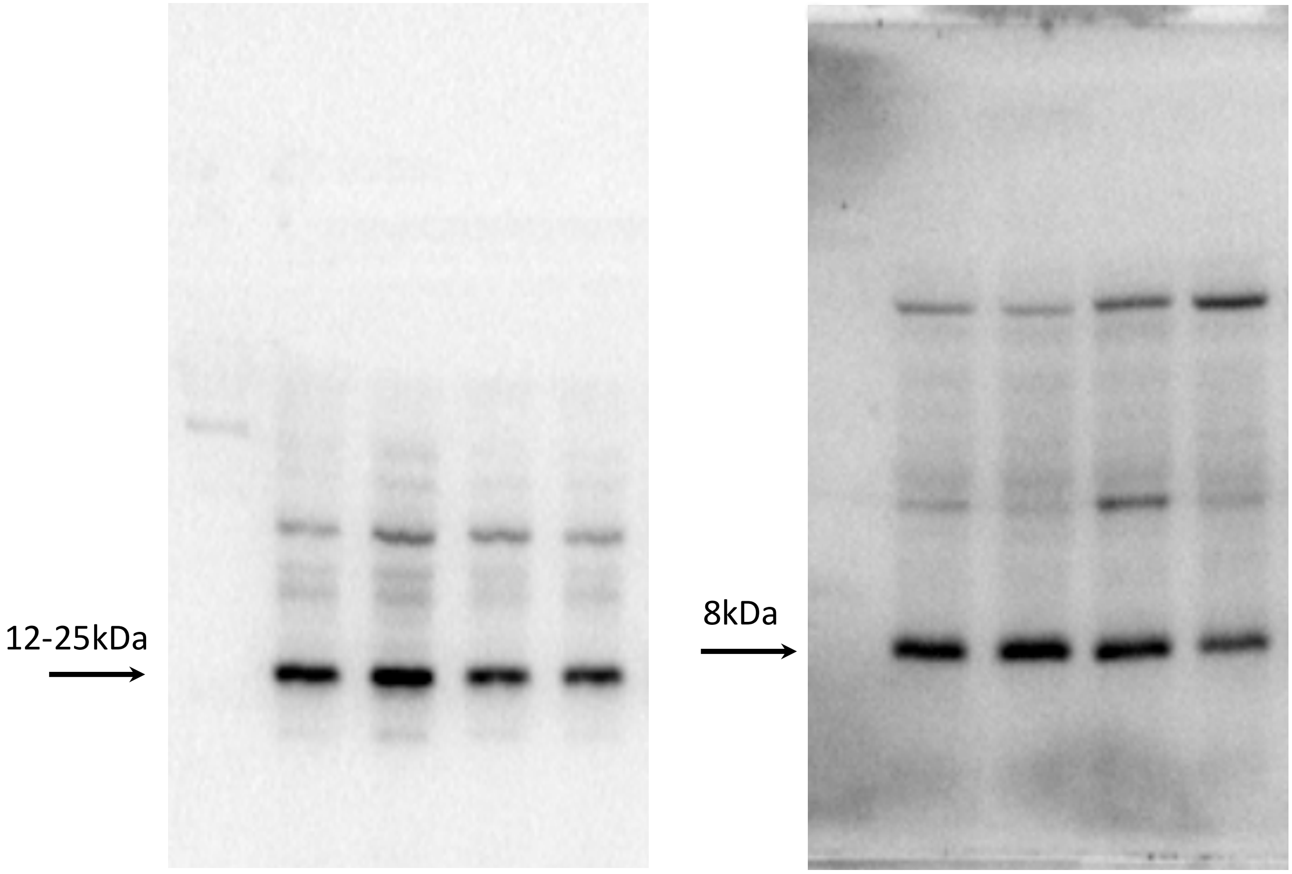

MCP-1 MIP-2

Supplementary figure S2: Uncropped images for MCP-1 and MIP-2.

Figure 8A

17kDa

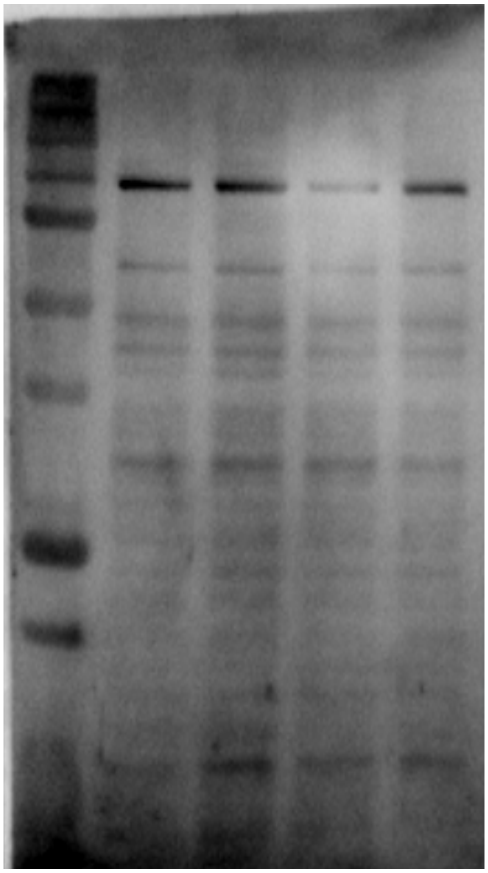

TNF-α

25kDa

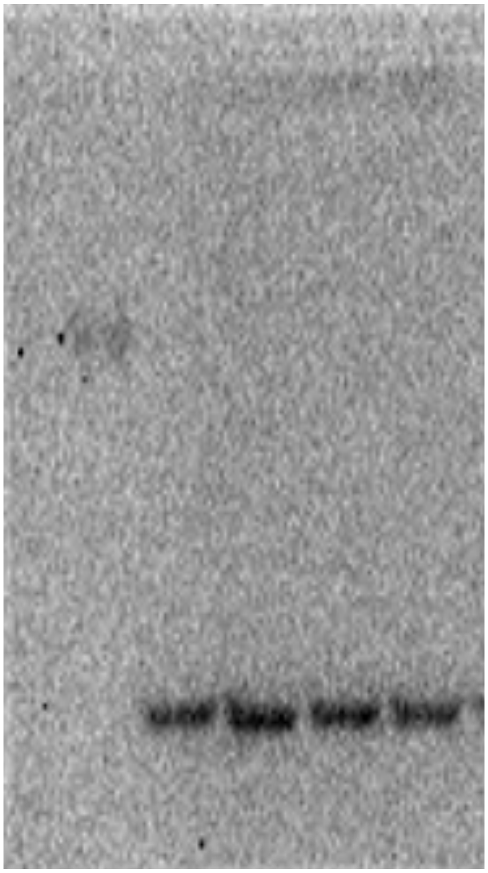

IL-6

Supplementary figure S3: Uncropped images for TNF-α and IL-6.

Figure 9A

25kDa

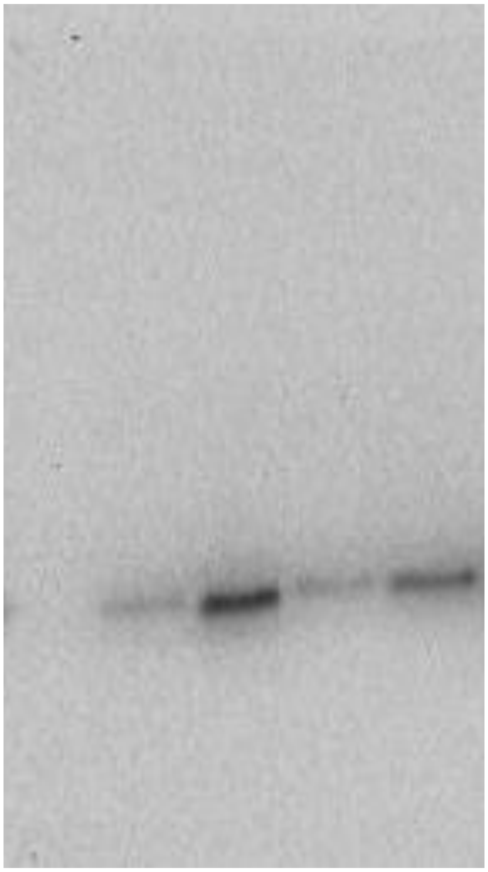

55-60kDa

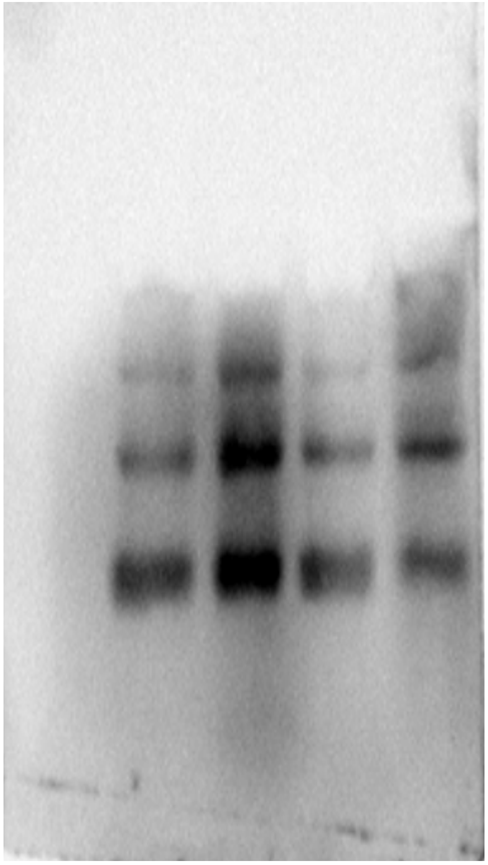

TGF-β

p-Smad2/3

Supplementary figure S4: Uncropped images for TGF-β and p-Smad2/3.

Figure 9C

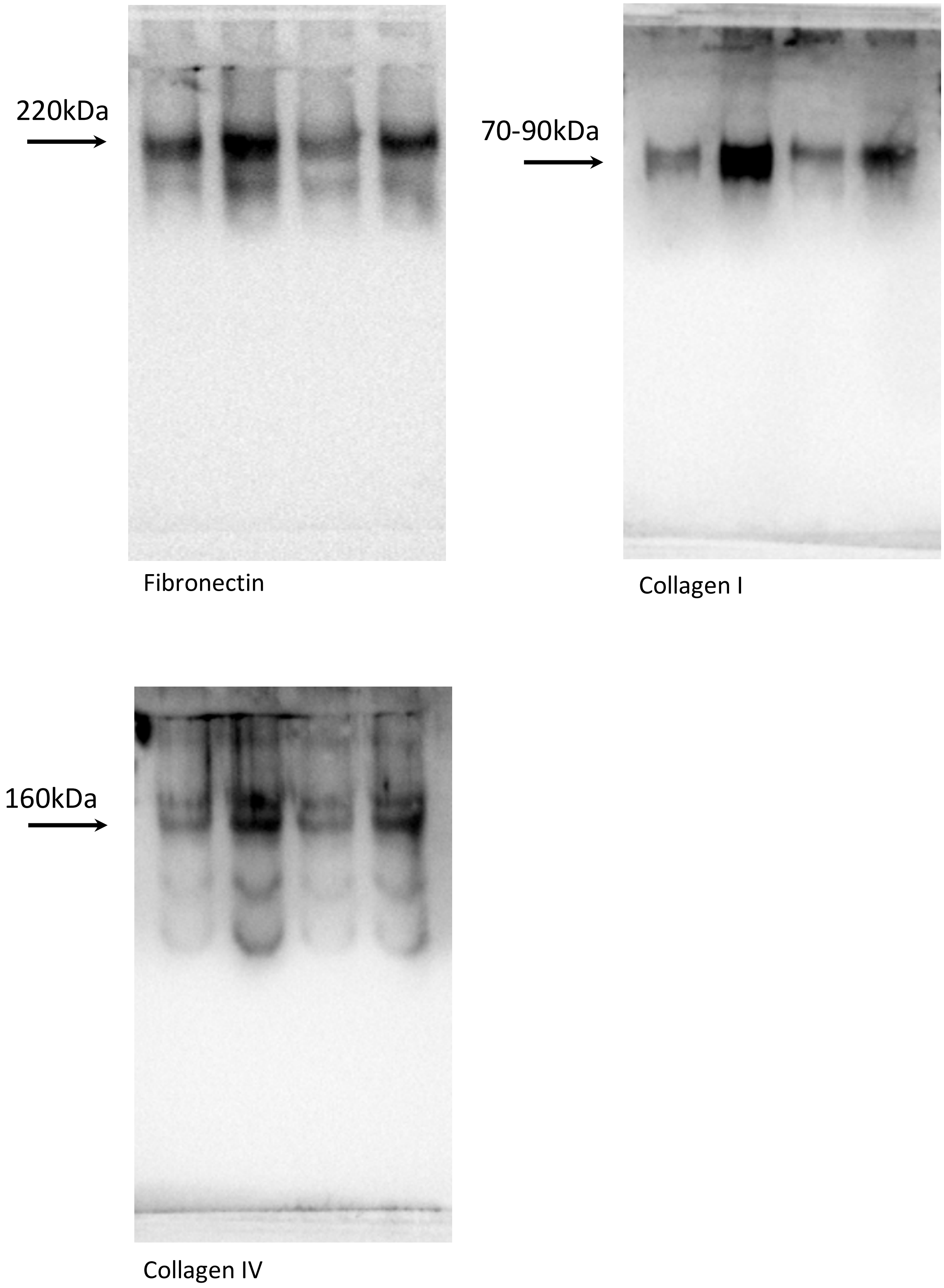

Supplementary figure S5: Uncropped images for fibronectin, collagen 1 and collagen IV.
